# Proteomic and transcriptomic analysis of selenium utilization in *Methanococcus maripaludis*

**DOI:** 10.1128/msystems.01338-23

**Published:** 2024-04-09

**Authors:** Katrina Funkner, Anja Poehlein, Nico Jehmlich, Richard Egelkamp, Rolf Daniel, Martin von Bergen, Michael Rother

**Affiliations:** 1 Faculty of Biology, Technische Universität Dresden, Dresden, Germany; 2 Genomic and Applied Microbiology & Göttingen Genomics Laboratory, Institute of Microbiology and Genetics, Georg-August-Universität Göttingen, Göttingen, Germany; 3 Department of Molecular Systems Biology, Helmholtz Centre for Environmental Research GmbH–UFZ, Leipzig, Germany; 4 German Centre for Integrative Biodiversity Research (iDiv) Halle-Jena-Leipzig, Leipzig, Germany; 5 University of Leipzig, Faculty of Life Sciences, Institute of Biochemistry, Leipzig, Germany; University of British Columbia, Vancouver, British Columbia, Canada

**Keywords:** selenium-dependent regulation, selenocysteine, *Archaea*, *Methanococcus maripaludis*, proteome, transcriptome

## Abstract

*Methanococcus maripaludis* utilizes selenocysteine- (Sec-) containing proteins (selenoproteins), mostly active in the organism’s primary energy metabolism, methanogenesis. During selenium depletion, *M. maripaludis* employs a set of enzymes containing cysteine (Cys) instead of Sec. The genes coding for these Sec-/Cys-containing isoforms were the only genes known of which expression is influenced by the selenium status of the cell. Using proteomics and transcriptomics, approx. 7% and 12%, respectively, of all genes/proteins were found differentially expressed/synthesized in response to the selenium supply. Some of the genes identified involve methanogenesis, nitrogenase functions, and putative transporters. An increase of transcript abundance for putative transporters under selenium depletion indicated the organism’s effort to tap into alternative sources of selenium. *M. maripaludis* is known to utilize selenite and dimethylselenide as selenium sources. To expand this list, a selenium-responsive reporter strain was assessed with nine other, environmentally relevant selenium species. While the effect of some was very similar to that of selenite, others were effectively utilized at lower concentrations. Conversely, selenate and seleno*-*amino acids were only utilized at unphysiologically high concentrations and two compounds were not utilized at all. To address the role of the selenium-regulated putative transporters, *M. maripaludis* mutant strains lacking one or two of the putative transporters were tested for the capability to utilize the different selenium species. Of the five putative transporters analyzed by loss-of-function mutagenesis, none appeared to be absolutely required for utilizing any of the selenium species tested, indicating they have redundant and/or overlapping specificities or are not dedicated selenium transporters.

**IMPORTANCE:**

While selenium metabolism in microorganisms has been studied intensively in the past, global gene expression approaches have not been employed so far. Furthermore, the use of different selenium sources, widely environmentally interconvertible via biotic and abiotic processes, was also not extensively studied before. *Methanococcus maripaludis* JJ is ideally suited for such analyses, thanks to its known selenium usage and available genetic tools. Thus, an overall view on the selenium regulon of *M. maripaludis* was obtained via transcriptomic and proteomic analyses, which inspired further experimentation. This led to demonstrating the use of selenium sources *M. maripaludis* was previously not known to employ. Also, an attempt—although so far unsuccessful—was made to pinpoint potential selenium transporter genes, in order to deepen our understanding of trace element utilization in this important model organism.

## INTRODUCTION

The trace element selenium is utilized by members of all three domains of life, Archaea, Bacteria, and Eukarya. Biologically active forms are the 2-selenouridine modification in certain transfer RNAs (1, 2), the dissociable cofactor in certain selenium-dependent molybdenum hydroxylases (3), and the selenium-containing metabolite selenoneine (4). The co-translationally inserted amino acid selenocysteine (Sec) is the most widely distributed biologically active form of selenium. However, the occurrence of Sec is unevenly distributed across the tree of life (5). Among the Archaea*,* three groups harbor Sec-containing proteins (selenoproteins): Methanococcales, Methanopyrales, and Lokiarchaeota (6, 7). Most archaeal selenoproteins are involved in methanogenesis, such as seven of the nine selenoproteins of the model methanogen *Methanococcus maripaludis* JJ (8). During selenium depletion, *M. maripaludis* is able to switch to a second set of enzymes, which include cysteine- (Cys-) containing isoforms of the selenoproteins (9, 10). Differential expression of genes for Sec-containing and Cys-containing isoforms, depending on the selenium availability, has, beyond *M. maripaludis*, also been observed in *Methanococcus voltae* (11) and in *Methanopyrus kandleri* (12). The selenoproteins of *M. maripaludis* JJ involved in methanogenesis are subunits of formate dehydrogenase (FdhA1 and FdhA2), formyl-methanofuran dehydrogenase (FwuB), heterodisulfide reductase (HdrA_U_), F_420_-dependent hydrogenase (FruA), and F_420_-independent hydrogenase (VhuD, VhuU) (8). For all methanogenesis-related selenoproteins, except for FdhA, Cys-containing isoforms can be synthesized by *M. maripaludis*, rendering growth on formate selenium dependent (9). Two further selenoproteins were identified via mutagenesis and ^75^Se labeling: selenophosphate synthetase (SPS) and HesB-like protein (13). While SPS is required for Sec synthesis, the function of the HesB-like protein is not clear.

The selenium-dependent switch from a selenoprotein to its Cys-containing isoform was shown to be mediated by regulation at the transcriptional level (10, 14). A LysR-type regulator designated HrsM acts as a transcriptional repressor for the Cys-encoding isogenes during selenium-adequate conditions by binding to the intergenic region between the *frc* and *vhc* operon (10, 15). However, how environmental selenium is sensed and how the signal(s) is/are transduced intracellularly through HrsM are unknown. It was suggested that SPS might play a role by providing selenophosphate that could serve as the physiological cue for the selenium status, as free selenide was ruled out (10).

So far, research on selenium-dependent regulation has focused on the Sec-/Cys-isoforms during selenium adequate/depleted conditions, leading to eight genes/proteins affected by the selenium availability in *M. maripaludis* JJ (16). In this study, we aimed to define the complete selenium regulon of *M. maripaludis* JJ, i.e., identify all proteins and genes of which the translation/expression is affected by selenium, via proteomic and transcriptomic analyses.

**Fig 1:**
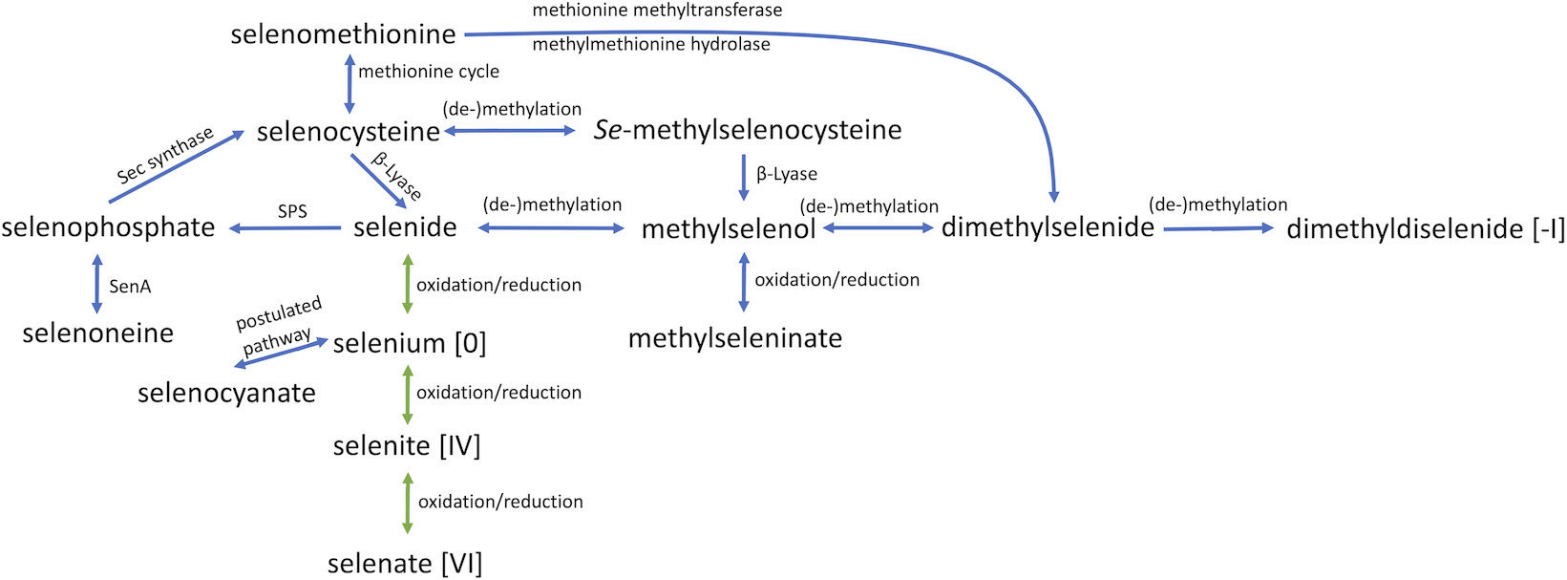
The selenium cycle. Green arrows indicate abiotic/microbial reactions. Blue arrows indicate microbial reactions. If no oxidation value for selenium is given it is [−II]; scheme adapted from (24).

*Methanococcus* species were shown to utilize two selenium species: (sodium) selenite and dimethylselenide (DMSe) (17). However, several metabolically and environmentally relevant selenium species exist, most of which are interconvertible, which could serve as selenium sources for *M. maripaludis* (Fig. 1). The selenium oxyanions selenate [oxidation state +VI], selenite [+IV], and selenide [−II] can be converted into each other, passing elemental selenium [0], by biotic or abiotic reduction/oxidation (18). As selenium is toxic for many organisms already at low concentrations, the element can be detoxified by methylation leading to the volatile species methylselenol (MSe), DMSe, and dimethyldiselenide (DMDSe) (19) (Fig. 1). For the cellular Se-compounds selenouridine, selenonenine, and Sec, selenophosphate synthesized by SPS from selenide and ATP is the precursor (20) (Fig. 1). For seleno-*DL*-methionine (SeMet), no specific biosynthetic pathway is known; in the presence of high selenium concentrations, plants and yeast incorporate selenium instead of sulfur in biosynthesis (21). To avoid Sec accumulation, some plant species are able to methylate Sec to *Se-*methylselenocysteine (MSec) (22). Other selenium species like selenocyanate (SeCN^−^) were found to be biogenically formed from selenate (23).

Due to the presence of the Cys-containing isoforms of the selenoproteins, hydrogenotrophic growth of *M. maripaludis* JJ is not impaired when selenium is scarce (9, 13, 25). Thus, the ability of the organism to utilize a certain selenium species cannot be easily assessed by growth experiments. To overcome this limitation and to generate an observable phenotype, expression of a gene encoding a β-lactamase (Bla), fused to the selenium-responsive *frcA* promoter, can be used as a proxy for selenium utilization (Fig. S1). When the cells are selenium replete, transcription of *frcA* and, consequently, synthesis and activity of Bla are minimal. Under selenium-depleted conditions, Bla activity is high (10). For any dissolved (i.e., non-gaseous) selenium species, the response of the reporter depends on (i) being transported into the cell, (ii) interacting productively with the selenium sensing/signal transduction machinery, and (iii) being a (direct or indirect) substrate for Sec synthesis. Intriguingly, no transmembrane transporter dedicated to and specific for any selenium species is known in any organism. The putative transmembrane protein complex YedEF co-occurs with SPS and was, therefore, suspected to be involved in the transport of selenium (26). As abundance of the *yedE*-encoding transcript, as well as those encoding other putative transporters, was increased upon selenium starvation, we hypothesized that *M. maripaludis* may try to increase its intracellular selenium level by increasing selenium transport (Fig. S2). To address this hypothesis, we conducted loss-of-function mutagenesis in the selenium reporter strain but found no clear evidence supporting it.

## RESULTS

To identify mRNAs and proteins of which the abundance was affected by the availability of selenium (using selenite as the “standard” selenium source) in *M. maripaludis*, a culture (C0), grown in the presence of 1 µmol L^−1^ selenite, was used to inoculate culture 1 (C1) with no selenium added to the media. This procedure was repeated seven times to create a dilution series of selenium in the cultures and to ensure that eventually carry-over of selenium and/or cellular storage could not affect the changes in the proteome/transcriptome over time. Comparisons were made by using C0 (selenium adequate) as reference and determine changes in C8 (selenium depleted) to the reference (fold change in C8 relative to C0).

### Proteome analysis

**Fig 2:**
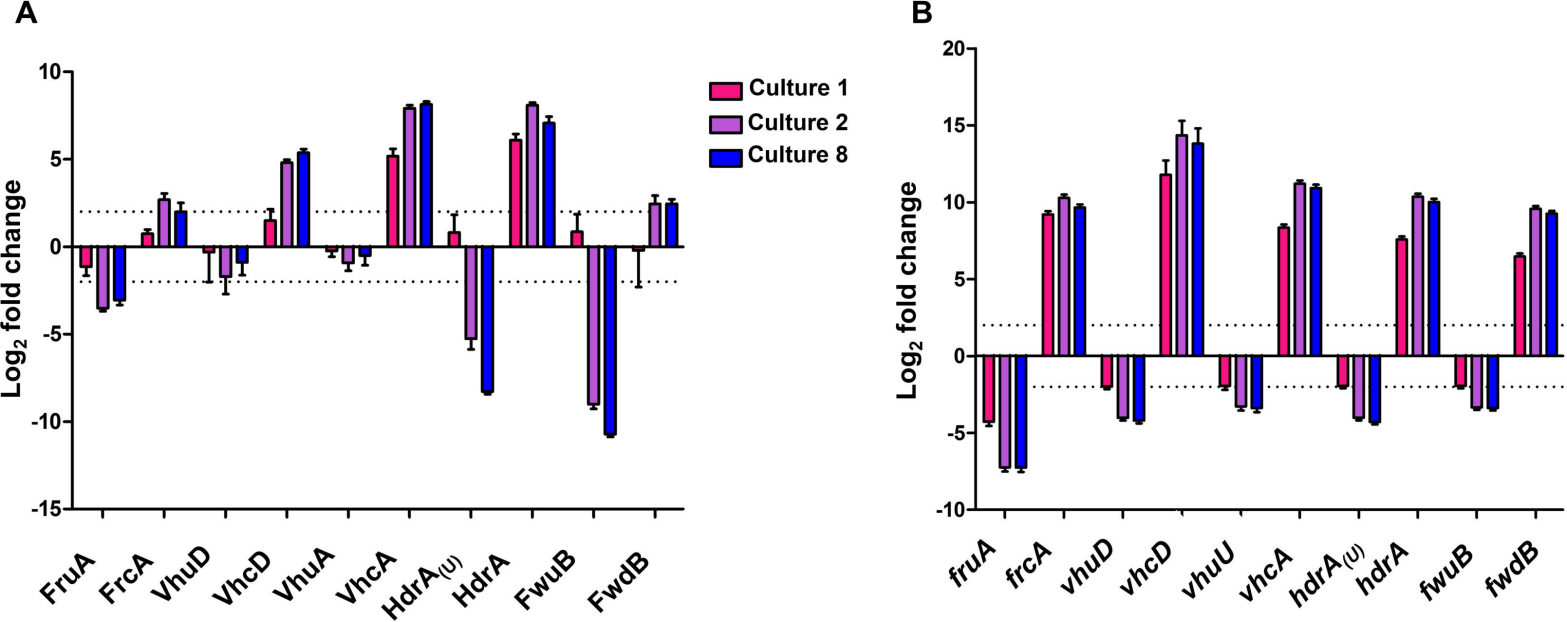
Se-dependent regulation of selenoproteins. (A) Log_2_-fold changes of selenoproteins involved in methanogenesis and their Cys-isoforms. Log_2_ fold changes are in comparison to culture 0 (selenium adequate), with culture 1 being the first grown in selenium-free media, culture 2 the second, and culture 8 the eighth. VhuU was not detected on proteomic level;instead, VhuA has been used as approximation. (B) Log_2_-fold changes of selenoprotein mRNAs involved in methanogenesis and their Cys-isoforms; note the difference in scale between panels A and B; dashed lines indicate the thresholds defined; shown are average values including the standard error of log_2_-fold change as error bars from five replicate cultures (see Materials and Methods).

The quality of the data was assessed though boxplot and principal component analysis (Fig. S3). Of the 1,864 predicted open reading frames (ORFs), 696 proteins (approx. 37%) were identified. Of those, 86 (12.4% of the detectable proteins, 4.6% of the encoded proteome) changed abundance in response to the selenium supply (Table S1). Of these 86 proteins, 38 showed lower abundances during selenium depletion, while 48 showed higher protein abundances (Table S1). A dilution effect for selenium was observed in C1, i.e., from 1 to (theoretically maximal) 0.05 µmol L^−1^ selenite; after C2, the proteome did not show selenium-dependent changes anymore, as exemplified by abundance of the five selenoproteins for which *M. maripaludis* synthesizes Cys-containing isoforms (Fig. 2A). During selenium depletion, abundances of selenoproteins and those of co-encoded subunits of the respective enzymes (Table S1) were reduced. The change ranged from −1.3 log_2_-fold for VhuA to −10.7 log_2_-fold for FwuB (Fig. 2A; Table S1), with VhuD and VhuA falling below the significance threshold. The change of the Cys-containing isoforms was more pronounced than that of the selenoproteins, with changes between +6.5 log_2_-fold for FrcA and +4.0 log_2_-fold for HdrA (Fig. 2A; Table S1). The different magnitudes of abundance change were due to the selenoproteins still being synthesized to some extent even in the absence of selenium, while most of the Cys-containing isoforms could not or barely be detected under selenium-adequate conditions (Table S2). Proteins involved in Sec synthesis and incorporation were either not detected or did not significantly (*P* value >> 0.05) change their abundance in response to the selenium supply.

Beside the Cys-containing isoforms being more abundant in selenium-depleted cells, two proteins identified are noteworthy: (i) the product of MMJJ_09780 (+4.4 log_2_-fold change), annotated as “phosphoglycerate transport regulatory protein PgtC precursor,” which is possibly part of an ABC transporter, and (ii) the product of MMJJ_15400 (+3.3 log_2_-fold change) annotated as selenium-binding protein. For the other proteins more abundant upon selenium depletion, no role in the metabolism of this element was obvious.

### Transcriptome analysis

**Fig 3:**
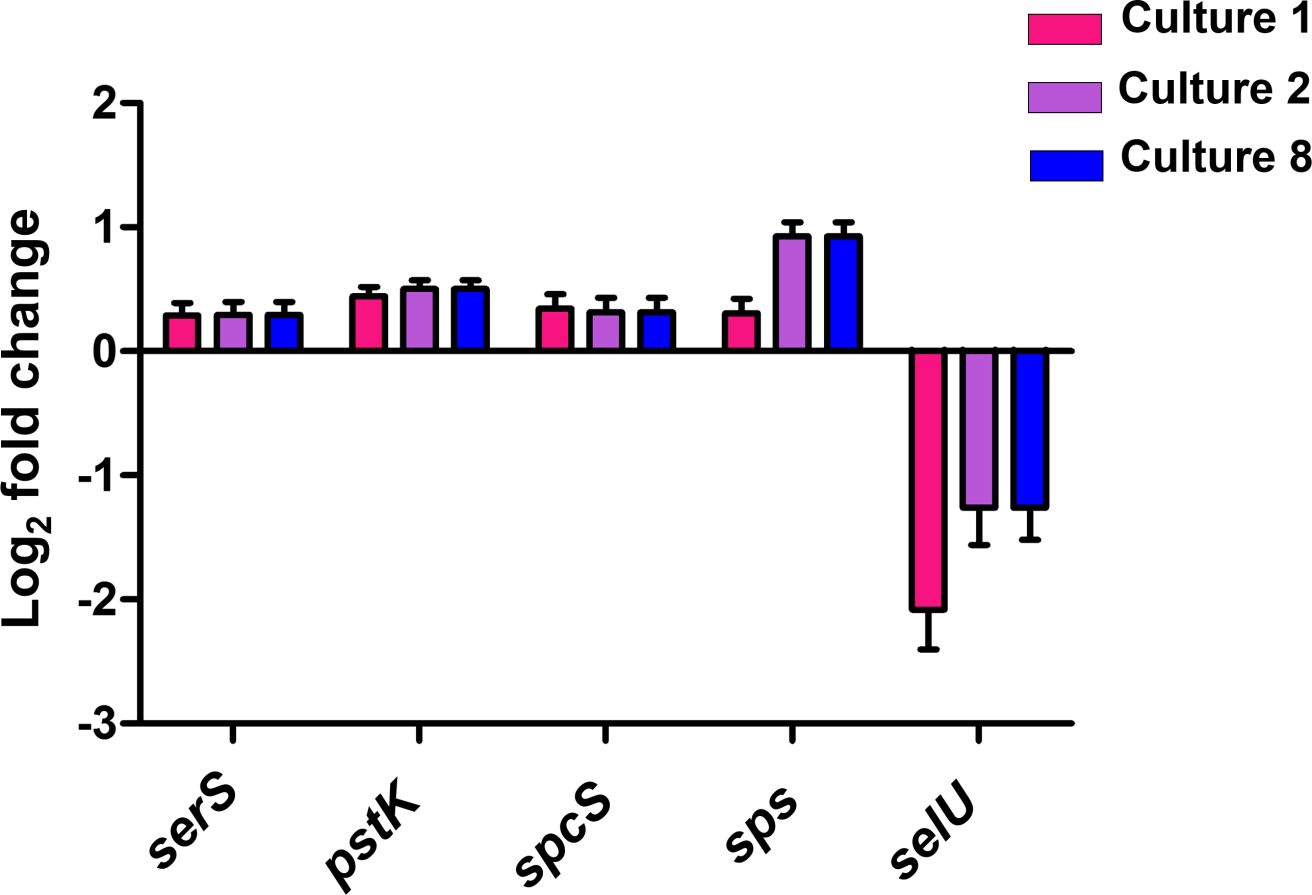
Se-dependent regulation of selenoprotein synthesis. Log_2_-fold changes of gene mRNAs involved in Sec-biosynthesis. Log_2_-fold changes are in comparison to culture 0 (selenium adequate), with culture 1 being the first grown in selenium-free media, culture 2 the second, and culture 8 the eighth; *serS*, gene for SerRS; *pstK*, gene for PstK; *selU*, gene for selenouridine synthase; *spcS*, gene for SepSecS; *selB* is not shown as *P*_adj_ > 0.05; shown are average values including the standard error of log_2_-fold change as error bars from five biological replicates (see Materials and Methods).

Only one-third of the encoded proteins was detected by our proteomic analysis, which is less than what was reported for *M. maripaludis* S2 (27). Furthermore, some of the Sec-/Cys-containing isoforms were not identified, as well as many membranous proteins. In order to increase the analyte pool for a more comprehensive view on the selenium regulon, a transcriptomic analysis was conducted. To ensure comparability of both data sets, the samples (cell pellets) were from the same cultures used for the proteomic analysis. The quality of the data was assessed though boxplot and principal component analysis (Fig. S3). Of the 1,864 predicted ORFs, 1,849 mRNAs (99.2%) were identified. The availability of selenium affected the abundance of a total of 126 (6.8%) of the detected mRNAs (Table S3). Under selenium-depleted conditions, mRNA transcripts of 51 genes (12 methanogenesis-related genes, 8 hypothetical genes, 10 putative transporter-encoding genes, and 21 other genes) were more abundant compared with selenium-adequate conditions. In general, the transcripts coding for both Cys-isoform hydrogenases and the Cys-isoform of the heterodisulfide reductase, as well as the formyl-methanofuran dehydrogenase (MMJJ_01770-800, *frcADGB*, MMJJ_01760-10, *vhcDGAB*, *hdrA*, and *fwdB*), showed the strongest increase during growth without selenium in comparison with growth with selenium. Their change varied between +8.3 log_2_-fold for *frcB* and +13.8 log_2_-fold for *vhcD* (Table S3). The abundance of transcripts of factors involved in Sec synthesis and incorporation, *serS*, *pstK*, *spcS* (encoding SepSecS), and *sps*, was not affected by the selenium status (Fig. 3, *selB* is not shown, due to a *P*_adj_ value of >>0.05). However, in C1, the abundance of *selU* (encoding selenouridine synthase) was decreased by over −2 log_2_-fold compared with C0 (Fig. 3). Ten putative transporter genes showed higher mRNA abundances during selenium depletion, when compared with selenium-adequate growth (Table 1). This included MMJJ_09780, already detected at the protein level (Table S1), with a change of +5.9 log_2_-fold. Other putative transporter-encoding genes with higher mRNA abundances were MMJJ_01610, (change of 6.3 log_2_-fold), MMJJ_00160 (change of 5.8 log_2_-fold), and MMJJ_13000 (change of 5.3 log_2_-fold) (Table 1).

**TABLE 1:** Genes for putative transporters, influenced by the selenium status

Locus tag^*a*^	Annotation	Log_2_-fold change	Deletion
MMJJ_01610	Putative sulfoacetate transporter SauU	+6.30 ± 0.13	JpST3
MMJJ_09780	Phosphoglycerate transport regulatory protein PgtC precursor	+5.88 ± 0.15	JpST1
MMJJ_00160	Molybdate ABC transporter periplasmic molybdate-binding protein	+5.79 ± 0.20	No
MMJJ_13000	YedE family protein	+5.28 ± 0.21	JpST5
MMJJ_09790	Cobalt transport protein	+4.18 ± 0.42	JpST1
MMJJ_09770	Putative 2-aminoethylphosphonate transport system permease protein PhnU	+4.13 ± 0.26	JpST1
MMJJ_04340	Inner membrane amino-acid ABC transporter permease protein YecS	+3.90 ± 0.39	No
MMJJ_00180	Putative permease	+3.10 ± 0.35	No
MMJJ_07110	Phosphate-binding protein PstS 1 precursor	+2.73 ± 0.20	JpST2
MMJJ_13030	Tripartite tricarboxylate transporter TctA family protein	+2.63 ± 0.55	No
MMJJ_04580	C4-dicarboxylic acid transporter DauA	+1.35 ± 0.22	JpST4
MMJJ_00280	Glycerol uptake facilitator protein	−3.72 ± 0.15	No
MMJJ_17760	Putative monovalent cation/H + antiporter subunit G	−3.10 ± 0.28	No
MMJJ_15410	Putative formate transporter 1	−2.43 ± 0.18	No
MMJJ_13580	Cobalt transport protein CbiM	−2.31 ± 0.14	No
MMJJ_13700	Putative monovalent cation/H + antiporter subunit E	−2.30 ± 0.24	No
MMJJ_17500	Phosphate-binding protein PstS one precursor	−2.28 ± 0.22	No

^*a*^ (8).

Under selenium-depleted conditions, mRNA transcripts of 75 genes showed lower mRNA abundances (13 methanogenesis-related genes, 20 hypothetical genes, 6 putative transporter-encoding genes, 8 nitrogen metabolism-related genes, and 28 other genes) in comparison to selenium adequate conditions (Table S3). The strongest decrease in mRNA transcript abundance was observed for the operon coding for the Sec-containing F_420_-reducing hydrogenase Fru (MMJJ_14570-14540, *fruADGB*) with changes between −5.0 log_2_-fold for *fruD* and −7.3 log_2_-fold for *fruA*. The operon coding for the Sec-containing F_420_-non-reducing hydrogenase Vhu, together with the Sec-containing heterodisulfide reductase and formyl-methanofuran dehydrogenase (MMJJ_11320-11380, *hdrA_U_*, *vhuDGAUB*, and *fwuB*), also showed a reduction of mRNA transcripts in selenium-depleted media. Their change varied between −3.4 log_2_-fold for *vhuU* and −4.3 log_2_-fold for *hdrA_U_*. Six putative transporter-encoding genes showed lower mRNA transcription abundances during selenium depletion, when compared with selenium-adequate growth. This included MMJJ_00280 (change of −3.7 log_2_-fold), MMJJ_17760 (change of −3.1 log_2_-fold), MMJJ_15410 (change of −2.4 log_2_-fold), and MMJJ_13580 (change of −2.3 log_2_-fold).

The transcriptomic response to selenium depletion was very similar to that observed at the proteome level, as exemplified by the abundance of the five selenoproteins for which *M. maripaludis* synthesizes Cys-containing isoforms (Fig. 2B). Like seen in the proteome, the change of the mRNA abundances for Cys-containing isoforms was more pronounced than that of the selenoprotein mRNAs, with changes between +9.3 log_2_-fold for *fwdB* and +11.0 log_2_-fold for *vhcA* (Fig. 2B; Table S3). The different magnitudes of abundance change were due to the selenoprotein genes still being transcribed to some extent even in the absence of selenium (between 16 ± 4 transcripts for *vhuU* and 3,821 ± 439 transcripts for *fwuB*), while for most of the genes for the Cys-containing isoforms, only few transcripts could be detected under selenium-adequate conditions (between 0.4 ± 0.5 reads for *vhcD* and 60.9 ± 7.6 reads for *hdrA*; Table S4).

Four other operons of which transcription was affected by the selenium status were as follows: (i) the *nif*-operon (MMJJ_01350-01420), with *nifl2* showing the strongest change in relative abundance (change of −3.5 log_2_-fold); (ii) an operon encoding unknown functions (MMJJ_16800-16910). It contains genes for homologs of HdrB/C, desulfoferrodoxin, ferritin, flavoprotein, divalent-cation tolerance protein, and a heavy metal transporter (changes for this operon varied between −2.1 log_2_-fold for CutA and −2.7 log_2_-fold for a DsrE/DsrF-like family protein-encoding transcript); (iii) transcripts for a sulfurtransferase system (TusAD, MJJ_07490-50) showed higher abundance during selenium depletion (4.5–4.6 log_2_-fold increase); and (iv) operon of MMJJ_01650-70 (+5.6–7.8 log_2_-fold change), annotated as “uroporphyrinogen decarboxylases,” but most probably part of the SdmABC methyltransfer system specific for methyl-selenides, first identified in *M. voltae* (17).

### Utilization of various selenium species

The increase of transporter-encoding transcripts upon selenium depletion might be regarded as a measure of *M. maripaludis* to tap into alternative selenium sources. To test for utilization of various selenium species (Table 2), the *M. maripaludis* reporter strain JPhydbla2, pre-grown in medium lacking selenium, was transferred into media containing various concentrations of the respective selenium species under study. The β-lactamase activity under acclimated conditions was determined from lysates after three subsequent transfers into identical media. If *M. maripaludis* is able to transport and utilize the respective selenium species, no β-lactamase will be synthesized, due to selenium-dependent repression of the *frcA* promoter controlling *bla* (10). If *M. maripaludis* is not able to transport or utilize the selenium respective species, the *frcA* promotor is derepressed leading to significant β-lactamase activity.

**TABLE 2:** Environmentally and metabolically available selenium species used in this study^*a*^

Selenium compounds (oxidation state)	Chemical formula	Abbreviation
Sodium selenate (VI)	Na_2_SeO_4_	Selenate
Sodium selenite (IV)	Na_2_SeO_3_	Selenite
Selenourea (−II)	(NH_2_)_2_CSe	SeUr
Seleno-*DL*-methionine (−II)	C_5_H_11_NO_2_Se	SeMet
Seleno-*L*-cystine (−II)	C_3_H_7_NO_2_Se	SecSec
*Se*-methylselenocysteine (−II)	C_4_H_9_NO_2_Se	MSec
Diphenyl diselenide (−II)	C_12_H_10_Se	DPDS
Potassium selenocyanate (−II)	K(SeCN)	SeCN
Methylseleninate (−II)	H_3_CSeO_2_^−^	MSA
Dimethylselenide (−II)	Se(CH_3_)_2_	DMSe
Dimethyldiselenide (−I)	Se_2_(CH_3_)_2_	DMDSe

^*a*^ (24, 28).

**Fig 4:**
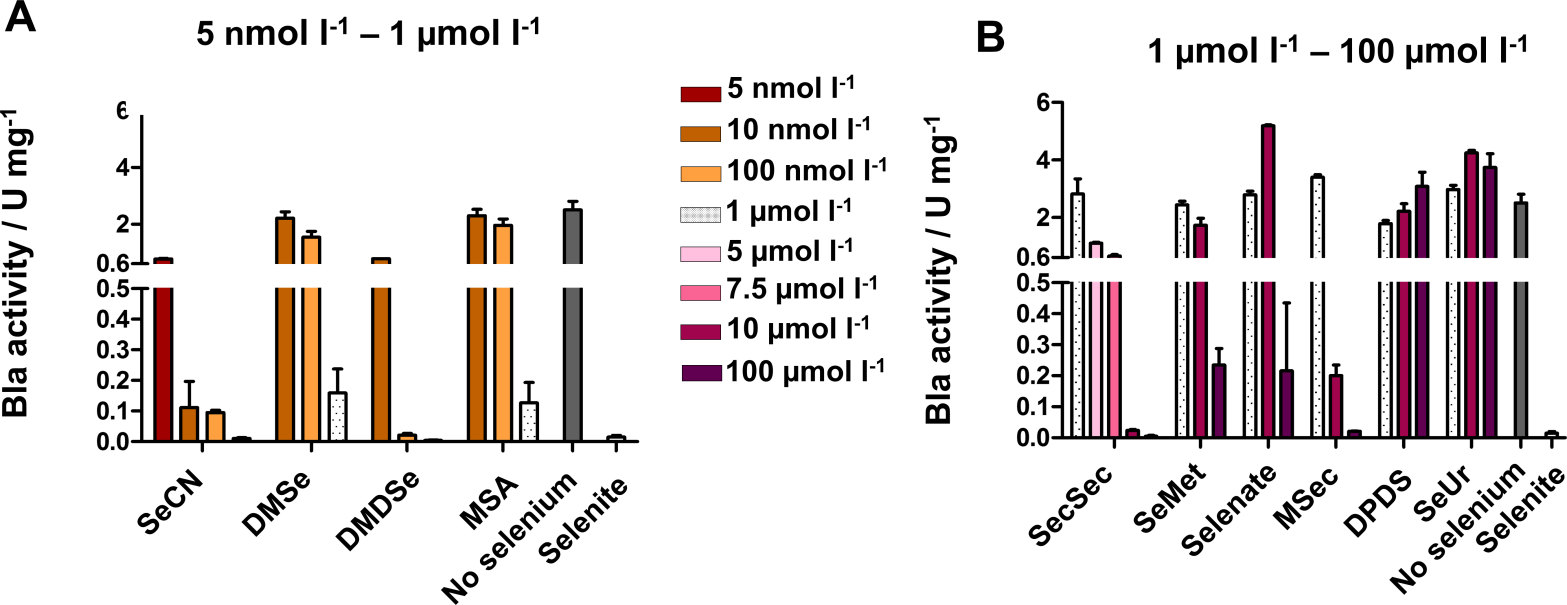
Utilization of various selenium species in *M. maripaludis* quantified via the selenium-responsive reporter as specific β-lactamase (Bla) activity (U mg^−1^). Selenium species (Table 2) were added to 5 nmol L^−1^, 10 nmol L^−1^, 100 nmol L^−1^, 1 µmol L^−1^, 5 µmol L^−1^, 7.5 µmol L^−1^, 10 µmol L^−1^, and 100 µmol L^−1^. (A) Selenium species utilized at 5 nmol L^−1^ (dark-brown), 10 nmol L^−1^ (light-brown), 100 nmol L^−1^ (orange), and 1 µmol L^−1^ (dotted), shown next to selenium-free media (gray) and sodium selenite at 1 µmol L^−1^. (B) Selenium species utilized at 1 µmol L^−1^ (dotted), 5 µmol L^−1^ (light-pink), 7.5 µmol L^−1^ (pink), 10 µmol L^−1^ (dark-pink), and 100 µmol L^−1^ (dark-purple), shown next to selenium-free media and selenite at 1 µmol L^−1^; shown are average values including the standard deviation as error bars from at least three biological replicates; the result shown was independently reproduced at least once.

The utilization of 11 different selenium species (Table 2) was tested at 5 nmol L^−1^, 10 nmol L^−1^, 100 nmol L^−1^, 1 µmol L^−1^, 5 µmol L^−1^, 7.5 µmol L^−1^, 10 µmol L^−1^, and 100 µmol L^−1^. None of these selenium species caused growth inhibition of the organism at the concentrations used (Fig. S4). The concentrations required to significantly reduce Bla activity (compared with the no selenium control) varied between the analyzed selenium sources (Fig. 4). Four of the tested species resulted in a selenium-adequate status (i.e., very low Bla activity) of JPhydbla2 at up to 1 µmol L^−1^ of the respective selenium source (Fig. 4A). These were selenocyanate (SeCN), DMDSe, DMSe and, methylseleninate (MSA). SeCN led to a low reporter activity (Table S5) already at 10 nmol L^−1^ and was, thus, utilized at approx. 10-fold lower concentration than selenite (Fig. 4A). For DMDSe, a concentration of 100 nmol L^−1^ in the media was sufficient to render the cells selenium replete (Fig. 4A; Table S5). DMSe and MSA both did not fully lead to selenium-adequate conditions at 1 µmol L^−1^ (Fig. 4A; Table S5). Four selenium species required higher concentrations to be “effectively” utilized by JPhydbla2: seleno-*L-*cystine (SecSec) led to reporter repression at 10 µmol L^−1^ but was still somewhat active with MSec as the selenium source (Fig. 4B; Table S6). With seleno-*DL*-methionine (SeMet) and sodium selenate, selenium-replete conditions were not even fully achieved at 100 µmol L^−1^ (Fig. 4B; Table S6). Two of the tested selenium species, selenourea (SeUr) and diphenyldiselenide (DPDS), led to no reduction of Bla activity in Jphydbla2 at any of the tested concentrations (Fig. 4B; Table S6). Thus, they were either not transported into or not utilized by the cells.

### Putative selenium transporter

The transcript abundance of several transporter-encoding genes changed upon selenium depletion (Table 1). Ten genes were apparently upregulated, and six were apparently downregulated. Our interpretation of this observation was that selenium depletion may lead in *M. maripaludis* to efforts to tap into alternative selenium sources by inducing transport functions. Downregulating a transporter may serve to limit unspecific loss of selenium from the cell. We chose to probe five of the upregulated loci, with no obvious function in energy metabolism (like nickel, iron, or molybdenum transport) by mutational analysis: MMJJ_09780, annotated as PgtC precursor; MMJJ_07110, annotated as PstS 1 precursor; MMJJ_01610, annotated as SauU; and MMJJ_13000, annotated as YedE. The fifth transporter, MMJJ_04580, was chosen based on its annotation as DauA/sulfate permease despite its mRNA abundance change falling below the significance threshold (Table 1).

For targeted mutagenesis of the putative transporter loci, a markerless disruption method for *Methanosarcina* (29) was adopted for use in *Methanococcus* (Fig. S5). Employing this method, the five transporter loci were deleted (Table 3). When co-encoded in an operon, the whole operon was deleted (Table 1).

**TABLE 3:** *M. maripaludis* strains used in this study

Strains	Description	Reference
JJ	Wild type	(30)
JPhydbla2	JJ, Δ*upt*::*PfrcA-bla*	(10)
JpST1	JPhydbla2, Δ*pgtC* (MMJJ_09760–90)::*frt*	This study
JpST2	JPhydbla2, Δ*pstS1* (MMJJ_07110–00) ::*frt*	This study
JpST3	JPhydbla2, Δ*sauU* (MMJJ_01610)::*frt*	This study
JpST4	JPhydbla2, Δ*dauA* (MMJJ_04580)::*frt*	This study
JpST5	JPhydbla2, Δ*yedE* (MMJJ_13000–10)::*frt*	This study
JpST1.3	JPhydbla2, Δ*pgtC* (MMJJ_09760–90)::*frt,* Δ*sauU* (MMJJ_01610)::*frt*	This study
JpST2.3	JPhydbla2, Δ*pstS1* (MMJJ_07110–00)::*frt,* Δ*sauU* (MMJJ_01610)::*frt*	This study
JpST4.3	JPhydbla2, Δ*dauA* (MMJJ_04580)::*frt,* Δ*sauU* (MMJJ_01610)::*frt*	This study
JpST5.3	JPhydbla2, Δ*yedE* (MMJJ_13000–10)::*frt,* Δ*sauU* (MMJJ_01610)::*frt*	This study

**Fig 5:**
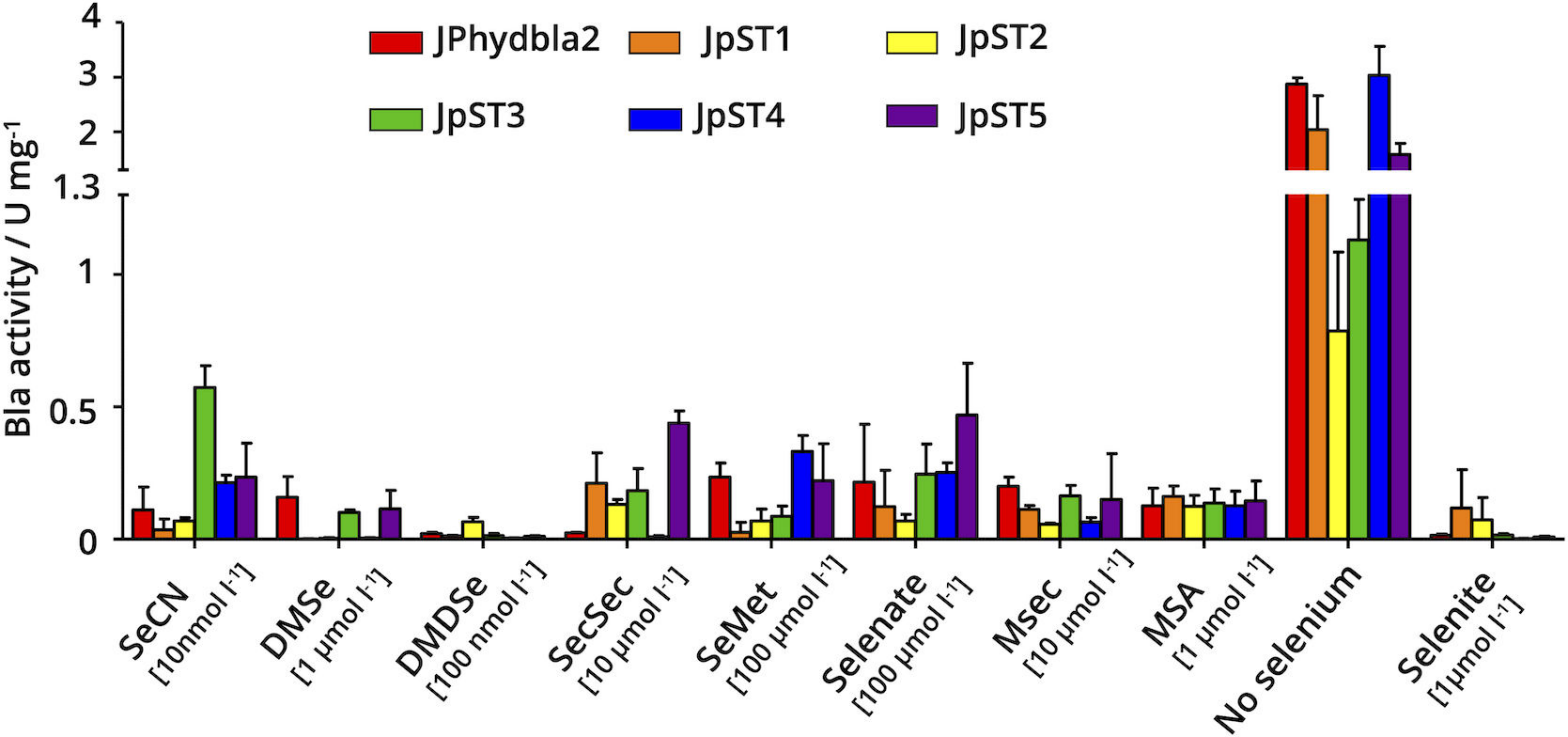
Utilization of various selenium species by mutant strains lacking putative transporters quantified via the selenium-responsive reporter as specific β-lactamase (Bla) activity (U mg^−1^). The lowest utilized concentration of the respective selenium species (see Fig. 4) was used. JPhydbla2 (red bars) was used as reference. JpST1 (orange bars), JpST2 (yellow bars), JpST3 (green bars), JpST4 (blue bars), and JpST5 (purple bars) were grown for 16–20 generations on the respective selenium species before the experiment; shown are average values including the standard deviation as error bars from at least three biological replicates; the result shown was independently reproduced at least once.

Strains JpST1 through JpST5 were tested for their ability to utilize the previously identified selenium species (Fig. 5). The strains were grown for three passages with the respective selenium species to establish acclimated conditions. The lowest possible concentration of the respective selenium species established for JPhydbla2 (Fig. 4) was used to make the response of the reporter readily observable. The Bla activities in the mutant strains were compared with those of JPhydbla2 (Fig. 5, red bars) grown with the same selenium species. If the transporter locus deleted in a strain was involved in the uptake of a certain selenium species, Bla activity in the strain grown with that species should be considerably higher than in the JPhydbla2 control. This was not the case for DMSe, DMDSe, SeMet, selenate, MSec, and MSA in any of the five mutant backgrounds (Fig. 5; Table S7). JpST3 exhibited a fivefold higher Bla activity on SeCN than JPhydbla2 (Fig. 5; Table S7), which could indicate that the SauU homolog encoded on MMJJ_01610 might be involved in the transport of that selenium species. A rise in Bla activity was also observed in JpST1, JpST2, JpST3, and JpST5 in the presence of SecSec. While the difference is between 18- and 60-fold, the still rather low values (Table S7) and the high concentration needed (10 µmol L^−1^) indicate that other routes for SecSec transport exist in *M. maripaludis*.

Double mutants containing a deletion in *sauU* and the four respective transporters were tested for their ability to utilize SeCN and 10 nmol L^−1^ each. The strains were grown for three passages with SeCN to establish acclimated conditions. No double mutant showed the same fivefold increase in Bla activity as JpST3 (Fig. S6). For JpST5.3 (carrying disruptions in *yedE* and *sauU*), a similar increase in Bla activity as for JpST3 was observed, while the other double mutants showed Bla activities not higher than JPhydbla2 (Fig. S6; Table S8).

## DISCUSSION

### Changes in mRNA and protein inventory upon selenium depletion

The proteomic and transcriptomic analyses both confirmed that transferring twice, 5% of a selenium-replete (1 µmol L^−1^) culture into medium to which no selenium source had been added, is sufficient to establish selenium-depleted conditions; further transfers did not alter the protein/mRNA inventory any further (Fig. 2). Selenium-dependent responses were still observed at C1, containing a theoretical maximum of 50 nmol L^−1^ selenium carried over from C0, which lies within the concentration range *M. maripaludis* senses and reacts to both on the transcriptional (10) and the translational (31) levels. C2 contains a theoretical maximum of 2.5 nmol L^−1^ selenium carried over from C1, which is below what is known to induce a response. Thus, *M. maripaludis* appears not to be able to accumulate significant amounts of selenium that could be mobilized during selenium depletion. We do not know why we identified in total fewer proteins than others (27).

The availability of selenium led to changes in the abundance of approx. 12% of the proteins detected by our analysis. Similar percentages have been observed upon H_2_-/nitrogen-/ and phosphate limitation in *M. maripaludis* (32). Deletion of Ehb hydrogenase changed the abundance of 10% of all detected proteins in comparison to the wild type (27). In *M. voltae*, iron and sulfur depletion affected the abundance of 40% of the proteome (33).

The abundance of many mRNAs and proteins changed when selenium was depleted. These changes were consistent with previous analyses or were expected to be regulated. Additionally, the current analysis confirmed co-regulation of subunits co-encoded in (putative) operons. Most prominent are the selenoproteins and their Cys-containing homologs (Fig. 2; Tables S1 and S3). Unexpected was the finding that genes encoded on a 10.2-kb region, previously shown (via overlapping reverse transcription PCR) to be co-transcribed (i.e., functionally linked) and involved in formate utilization (34), appear to be differentially regulated. The observed drop in the abundance of the subunits of Sec-containing formate dehydrogenase (Fdh) (MMJJ_15440, MMJJ_15460) reflects the selenium-depleted status of the cells unable to insert Sec, despite transcript levels of the encoding genes for the subunits remaining constantly high, even when the pathway for Sec biosynthesis is disrupted (25). The constitutive expression of Fdh-encoding genes probably enables the organism, lacking a Cys-isoform, to rapidly synthesize this important enzyme when selenium becomes available. Conversely, other formate-related genes in that genomic region, like the genes encoding a selenium-binding protein [MMJJ_15400 (35)], and FdhC, a putative formate transporter (MMJJ_15410), are clearly downregulated upon selenium depletion (−2.4 and −2.7 log_2_-fold, respectively) indicating that the promoters in this region were incompletely mapped (34).

Transcript levels of the other selenoprotein genes did not drop under selenium-limited condition to the same extent the Cys-encoding isogenes did when selenium was ample. The same was true for the respective proteins, indicating that *M. maripaludis* prefers to use the selenoproteins, thereby accepting to “sacrifice” resources (for transcribing the genes, for synthesizing truncated proteins, and for degrading them) during selenium depletion, in favor of their superior efficiency under selenium-replete conditions (9). Another factor illustrating selenium-dependent adaptations in energy metabolism of *M. maripaludis* was the [Fe]-hydrogenase Hmd (36). Both the gene for the apoenzyme (MMJJ_09590) and those for some of the enzymes synthesizing Hmd’s iron guanylyl pyridone cofactor, HcgA and HcgG (MMJJ_09600-10) (37) were upregulated (*hmd* +5.2 log_2_-fold, *hcgA* +4.2 log_2_-fold, and *hcgG* +3.9 log_2_-fold) upon selenium depletion, possibly to compensate for diminished levels of reduced cofactor F_420_ (F_420_H_2_), due to reduced F_420_-dependent hydrogenase activity (a selenoprotein). When Hmd is present, it can replace the F_420_-dependent methylene-tetrahydromethanopterin dehydrogenase (Mtd, MMJJ_07050,–3.8 log_2_-fold change), leaving only one, rather than two reactions in the pathway F_420_H_2_ dependent (38).

Selenoprotein synthesis itself (i.e., factors involved in Sec synthesis and incorporation) appears not influenced by the selenium status of the cell, as all transcript and protein abundances stay constant during selenium depletion. This includes (at the transcriptional level) *sps*, itself a selenoprotein. A constant transcription of the Sec-biosynthesis genes further indicates a preference of the Sec-containing proteins, as a rapid reaction mechanism toward supplemented selenium.

Other genes influenced by the selenium supply are possibly associated with nitrogen fixation (MMJJ_01380-420, MMJJ_05800, MMJJ_05840; Table S3). It is not clear why these *nif* mRNAs would be reduced in abundance upon selenium depletion, as the media used contain ample amounts of ammonia, making this energetically costly metabolism (39) unnecessary. Previous studies suggested reduction of non-physiological substrates by nitrogenases, like azides, cyanides, CO, or CO_2_. Those substrates are assumed to follow a more complex reduction pathway, and some of them can be ultimately reduced to methane or formate (40). Such side activity in *M. maripaludis* might be unfavorable during selenium depletion, as the FDH (a selenoproteins) is not available. Another possibility for a link between selenium and diazotrophy could be the HesB-like selenoprotein (MMJJ_08070). It is distantly related to IscA, which is possibly involved in iron-sulfur cluster assembly and often co-transcribed with *nif* genes (41). Loss of *hesB-like* homologs impaired growth during nitrogen limitation in certain bacteria (42, 43). However, a disruption mutant in *M. maripaludis* (13) was not impaired in its ability to fix nitrogen (Quitzke and Rother, unpublished observation).

Transcripts for subunits A and D of the sulfurtransferase system Tus were more abundant during selenium depletion (MMJJ_07490-50). The TusABCD complex mediates the 2-thiouridylation of tRNA modification 5-methylaminomethyl-2-thiouridine (mnm^5^s^2^U) (44). Why *M. maripaludis* might require more thiouridine/mnm^5^s^2^U-modified tRNAs during selenium depletion is not obvious. Also affected by the selenium supply were methanogenesis marker proteins (MMPs) 4 (MMJJ_14930) and 14 (MMJJ_16220). MMPs are conserved in, and largely restricted to, methanogenic archaea but mostly uncharacterized.

### *M. maripaludis* utilizes novel selenium species

It was unknown whether *Methanococcus* species could utilize other (beside selenite and DMSe) environmentally relevant selenium species. To investigate this question without a clear “depletion phenotype” (caused by the Cys-isoforms), we made use of a selenium-responsive reporter system, harboring the gene of β-lactamase under the control of the *frcA* promoter. Of the 10 tested selenium species, most of which are transformed into each other in the environment (Fig. 1), SeCN and DMDSe were utilized at lower concentrations than selenite. DMDSe is produced by microbes, plants and animals as a selenium detoxification mechanism (45, 46), while SeCN can be produced by some fresh water green algae (23).

DMSe has been identified as a selenium source for *M. voltae* during starvation of selenium salts (17). A methyltransferase system consisting of a corrinoid protein (SdmA), a substrate-specific corrinoid methyltransferase (SmdB), and a coenzyme M methyltransferase (SdmC) was linked to mobilization of this selenium source; a very similar locus (MMJJ_01670-50) was highly expressed when *M. maripaludis* starved for selenium (Table S3). A second methyltransferase gene in *M. maripaludis*, MMJJ_16960, with a putative upstream corrinoid protein-encoding gene, was also highly expressed under selenium-depleted conditions (Table S3), suggesting that it functions in DMDSe activation. Both DMSe and DMDSe are volatile and assumed not to require active transport into the cell (19). Still, the minimal concentrations required of both species for effective utilization may argue for an active transport system to increase cellular availability at small environmental concentrations.

Interestingly, seleno-modified amino acids were not readily available for *M. maripaludis*. Rather non-physiological concentrations of 10–100 µmol L^−1^ were needed for seleno-*L*-cystine, seleno-*DL*-methionine, or *Se*-methylselenocysteine to be utilized (Fig. 4B). Chemolithoautotrophic organisms often lack transport systems for organic molecules, including amino acids, resulting in ineffective uptake (47). However, *M. maripaludis* was shown to transport alanine and use it as a nitrogen source (48). Our data suggest that the available inventory of transporters is not suited for seleno-*L*-cystine, seleno-*DL*-methionine, and *Se*-methylselenocysteine and that *M. maripaludis* might not utilize selenium when it is bound to amino acids as a selenium source in its natural environment. Intriguingly, selenate required a concentration of 100 µmol L^−1^ to be utilized. Selenate can be abiotically reduced to selenite, which *M. maripaludis* utilized at 1,000-fold lower concentration. Although the selenate/selenite reduction potential is >0.4 V at pH 7 (49), the data argue for only limited reduction of selenate in McSe medium.

### Active selenium transport?

To our knowledge, no specific selenium transporter has been identified so far. The putative transporter genes with higher mRNA abundances during selenium depletion might indicate such an ability of *M. maripaludis*. Five promising loci were deleted from the chromosome of the JPhydbla2 reporter strain by an adapted markerless disruption method and analyzed for their capability to transport the newly identified selenium species.

Of the five that were tested, no single transporter could be identified to be specific for any of the 10 selenium species used. The only significant uptake phenotype was observed for SauU and SeCN at 10 nmol L^−1^ (Fig. 5). A sulfite transporter in yeast (not occurring in *M. maripaludis*) was shown to confer selenium tolerance (50). A mild transport phenotype was also observed for JpST1, JpST2, JpST3, and JpST5 with the substrate SecSec, indicating they might translocate this oxidized di-amino acid with low specificity. While phosphate and phosphoglcerate transport was attributed to the Pst and Pgt system, respectively (51, 52), the putative transmembrane YedEF system has been proposed to be involved in selenium metabolism, as it co-occurs with SPS (26). Due to the high concentration required for utilization of SecSec, unspecific co-transport seems likely. None of the tested putative transporters could be unequivocally linked to any of the selenium species tested in this study. Besides the obvious reason that the transporters analyzed simply do not transport selenium species, redundancy in (unspecific) activity of more than one of the transporters is feasible. With the loss of one, *M. maripaludis* would still be able to use others and no loss in selenium uptake capabilities would be observable in our experimental set-up. To approach this issue, the markerless disruption system was devised. However, none of the tested double mutants lacking *sauU* and any of the other four tested transporter genes showed the same or a stronger deficit in selenium transport then JpST3 (Fig. S6), except JpST5.3. The fact that the other double mutants approached the wild type, i.e., “lost” their transport phenotype, indicates that there might be regulatory “interaction” between the individual transporters, either at the level of gene expression (i.e., loss of one would increase another) or at the protein level or both. Such genetic interactions were observed for regulation of methyl-sulfide- and for methanol-specific methyltransferases in *Methanosarcina acetivorans* (53, 54).

### Conclusions

This study aimed to investigate the selenium regulon in *M. maripaludis* JJ. We identified 86 proteins and 126 transcripts which show selenium-dependent abundance, including the Sec-/Cys-containing isoenzymes involved in methanogenesis. Due to constitutive, i.e., selenium-independent, expression of Sec biosynthetic genes and those encoding Sec-dependent formate dehydrogenase, along with the less stringent downregulation of the selenoprotein genes, we assert a strong preference in *M. maripaludis* for using the selenoproteins over their Cys-containing isoforms. Two of the previously uncharacterized selenium species *M. maripaludis* utilized at similar or lower concentrations as selenite. On the other hand, seleno-amino acids are not effective sources of selenium for *M. maripaludis*. Lastly, of five putative selenium transporters examined through loss-of-function mutagenesis, none appeared to be required for utilization of any of the tested selenium species.

## MATERIALS AND METHODS

### Strains, media, growth, and Bla assay

*Escherichia coli* strain DH10B was grown under standard conditions (55). For plasmid selection after electroporation (56), 100 µg mL^−1^ ampicillin was added to the media. *Methanococcus maripaludis* JJ [DSMZ 2067 (30) and its derivatives (Table 3)] was cultivated in McSe medium (9), lacking sodium sulfide but containing 2 g L^−1^ *L*-cysteine (solid added during media preparation) and 10 mmol L^−1^ sodium acetate. When needed, 2.5 µg mL^−1^ puromycin (Merck, Darmstadt, Germany) or 0.25 mg mL^−1^ 6-azauracil (6AU) (Merck, Darmstadt, Germany) was added from sterile anaerobic stocks. Either 26-mL glass culture (Balch) tubes (Ochs, Bovenden, Germany) containing 5 mL or 100-mL serum bottles (Ochs) containing 25 mL media were used. Cultures were pressurized once with 1.5 × 10^5^ Pa of H_2_:CO_2_ (80:20) which served as the sole energy source and incubated at 37°C with gentle agitation in a rack shaker (Kühner shaker, Basel, Switzerland) at 110 rpm or a New Brunswick TC-7 Culture Roller Rotator (Eppendorf, Hamburg, Germany) at 8 rpm. Growth was monitored photometrically as optical density at 578 nm (OD_578_) using a Genesys 20 spectrophotometer (Themo Fisher Scientific).

For selenium-adequate conditions, 1 µmol L^−1^ sodium selenite was added to the media from a sterile anaerobic stock. Stocks of other selenium species (Table 2) were prepared with appropriate anaerobic solvents [H_2_O, 70% (vol/vol) ethanol, or 0.5 mol L^−1^ HCl] and sterilized by filtration (0.45 µm, Filtropur S, Sarstedt, Germany). For assessing utilization of various selenium species in *M. maripaludis in vivo*, a selenium-responsive reporter system was employed. All analyzed strains are based on JPhydbla2 (10), which carries a codon-optimized gene for β-lactamase (of *E. coli*) under the control of the *frcA* promoter in the *upt* locus (Fig. S1). JPhydbla2 was pre-grown in McSe medium (without selenium), followed by growth in McSe containing the respective selenium species at various calculated concentrations (100 µmol L^−1^, 10 µmol L^−1^, 7.5 µmol L^−1^, 5 µmol L^−1^, 1 µmol L^−1^, 100 nmol L^−1^, 10 nmol L^−1^, and 5 nmol L^−1^) for three passages (i.e., 16–20 generations in total), after which cells were considered acclimated (i.e., at a “steady state”) with regard to the selenium source in question.

To quantify Bla reporter activity, strains were grown in the respective media to an OD_578_ of 0.5 ± 0.02 followed by harvesting the cells by centrifugation at 5,000 × *g* for 10 min. Cell lysis, sample preparation, recording of initial rate of nitrocefin hydrolysis, determination of protein concentration, and calculation of the specific Bla activities (1 U = 1 µmol nitrocefin cleaved per min) were conducted as described (57), except no promoter-less construct was used for correcting values. Experiments were conducted in triplicates and repeated at least once.

### Molecular methods and cloning

Plasmids used in this study are listed in Table S9. All DNA fragments used for cloning were derived from PCR (oligonucleotides used are listed in Table S10) or were commercially synthesized (Biocat, Heidelberg, Germany). Cloned PCR fragments were verified by Sanger sequencing using the BigDye Terminator Cycle Sequencing protocol (Microsynth Seqlab, Göttingen, Germany).

For targeted mutagenesis, a markerless gene disruption method for *Methanosarcina* (29, 58) was adopted to *Methanococcus* (Fig. S5). This method is based on double homologous recombination of a linearized DNA fragment of plasmid pMMD1 (plasmid *Methanococcus* Markerless Disruption) (Fig. S7) into the genome of a *M. maripaludis* strain lacking the *upt* gene (MMJJ_02980), thereby replacing the gene of interest by a selection/counter-selection cassette (Fig. S7). Recombination is enabled by sequences of the up- and downstream regions bordering the cassette, which consists of a codon-optimized allele of *pac* (N*-pac*), conferring resistance toward puromycin (15) and *upt*, which causes sensitivity toward 6AU (in a 6AU-resistant Δ*upt* strain). The cassette is flanked by two Flp recombinase recognition target (FRT) sites (Fig. S7), allowing for its removal by site-specific recombination through Flp recombinase (59). The codon-optimized *flp* is encoded on the helper plasmid pFlpMM (plasmid Flp recombinase *Methanococcus Maripaludis*), (Fig. S8) under control of the strong constitutive *hmvA* promoter of (60).

Transformation and plating of *M. maripaludis* were conducted as described (31). Genotypes of *M. maripaludis* mutant strains were verified by sequencing of PCR products spanning the altered region.

### Proteome and transcriptome analyses

*M. maripaludis* JJ was grown in five replicate cultures in McSe medium containing 1 µmol L^−1^ sodium selenite. At OD_578_ ≈ 0.5, 1- and 2-mL samples were harvested by centrifugation and the cell pellets were snap frozen in liquid nitrogen and stored at −80°C until used (culture 0, C0). Concomitantly, five fresh cultures were inoculated 1:20 into McSe media without selenium (culture 1, C1). This process was repeated seven more times, leading to nine sampling timepoints in total (culture 0, C0–culture 8, C8).

For protein analysis, cell pellets were dissolved in lysis buffer (8 M urea, 2 M thiourea, 1 mmol L^-1^ phenylmethylsulfonyl fluoride) and incubated for 30 min at 95°C while shaking at 1,400 rpm (Thermomixer, Eppendorf, Hamburg, Germany), followed by 3 min of ultrasonication (three cycles, 80% amplitude, 30 sec intervals) in a cold water bath. After centrifugation, 6.75 µL of 2.5 mmol L^-1^ 1,4-dithiothreitol (in 20 mmol L^-1^ ammonium bicarbonate) were added to the resulting pellets and incubated for 1 h at 60°C while shaking at 1,400 rpm. Subsequently, 150 µL iodoacetamide (10 mmol L^-1^ in 20 mmol L^-1^ ammonium bicarbonate) were added and incubated for 30 min at 37°C while shaking at 1,400 rpm in the dark. Two hundred microliters of 20 mmol L^−1^ ammonium bicarbonate was added, and the protein lysates were proteolytically cleaved overnight at 37°C with trypsin (2.5 µL of 0.1 µg µL^−1^ trypsin, Promega). Proteolysis was stopped by adding 50 µL 10% formic acid. The peptide lysates were desalted using ZipTip μC18 tips (Merck Millipore, Darmstadt, Germany) and resuspended in 15 µL 0.1% formic acid, before separated by nano high-performance liquid chromatography mass spectrometry (UltiMate 3000 RSLCnano, Dionex, Thermo Fisher Scientific). Mass spectrometric analyses of peptide lysates were performed on a Q Exactive HF mass spectrometer (Thermo Fisher Scientific) coupled with a TriVersa NanoMate (Advion Ltd., Harlow, UK). Samples were injected on a trapping column (Acclaim PepMap 100 C18, 3 µm, nanoViper, 75 µm × 2 cm, Thermo Fisher Scientific) with 5 µL min^−1^ by using 98% water/2% acetonitrile/0.5% trifluoroacetic acid and separated on an analytical column (Acclaim PepMap 100 C18, 3 µm, nanoViper, 75 µm × 25 cm, Thermo Fisher Scientific) with a flow rate of 300 nL min^−1^. The mobile phase was 0.1% formic acid in water (A) and 80% acetonitrile/0.08% formic acid in water (B). Full MS spectra (350–1,550 *m/z*) were acquired in the Orbitrap at a resolution of 120,000 with an automatic gain control (AGC) target value of 3 × 10^6^ ions.

Acquired mass spectrometrydata were analyzed with the Proteome Discoverer (v.2.5, Thermo Fisher Scientific) employing SEQUEST HT. Protein identification was performed using a database generated from the published genome sequence (GenBank accession No. CP026606) and common laboratory contaminations. Searches were conducted with the following parameters: trypsin as enzyme specificity and two missed cleavages allowed. A peptide ion tolerance of 10 ppm and an tandem mass spectrometry (MS/MS) tolerance of 0.02 Da were used. As modifications, oxidation (methionine) and carbamidomethylation (cysteine) were selected. Peptides that scored a *q*-value > 1% based on a decoy database and with a peptide rank of 1 were considered identified. Differential abundances were defined as significant with a threshold of ±1.325 log_2_-fold difference.

The mass spectrometry proteomics data have been deposited to the ProteomeXchange Consortium via the PRIDE (61) partner repository with the data set identifier PXD047095.

Transcriptomic analysis was conducted using the same samples as in the proteomic analysis, as a way to ensure their comparability. Harvested cells were re-suspended in 800 µL RLT buffer (RNeasy Mini Kit, Qiagen) with β-mercaptoethanol (10 µL mL^−1^), and cell lysis was performed using a laboratory ball mill. Subsequently, 400 µL RLT buffer and 1,200 µL 96% (vol/vol) ethanol were added. For RNA isolation, the RNeasy Mini Kit (Qiagen) was used as recommended by the manufacturer, but instead of RW1 buffer, RWT buffer (Qiagen) was used in order to isolate RNAs smaller than 200 nucleotides also. To determine the RNA integrity number, the isolated RNA was run on an Agilent Bioanalyzer 2100 using an Agilent RNA 6000 Nano Kit as recommended by the manufacturer (Agilent Technologies, Waldbronn, Germany). The remaining genomic DNA was removed by digesting with TURBO DNase (Invitrogen, Thermo Fisher Scientific, Paisley, UK). The Illumina Ribo-Zero plus rRNA Depletion Kit (Illumina Inc., San Diego, CA, USA) was used to reduce the amount of rRNA-derived sequences. For sequencing, the strand-specific cDNA libraries were constructed with a NEB Next Ultra II Directional RNA Library Preparation Kit for Illumina and the NEB Next Multiplex Oligos for Illumina (96) (New England BioLabs, Frankfurt am Main, Germany). To assess the quality and size of the libraries, samples were run on an Agilent Bioanalyzer 2100 using an Agilent High Sensitivity DNA Kit as recommended by the manufacturer (Agilent Technologies). The concentration of the libraries was determined using the Qubit dsDNA HS Assay Kit as recommended by the manufacturer (Life Technologies GmbH, Darmstadt, Germany). Sequencing was performed on the NovaSeq 6000 instrument (Illumina Inc., San Diego, CA, USA) using a NovaSeq 6000 SP Reagent Kit (100 cycles) and the NovaSeq XP 2-Lane Kit v1.5 for sequencing in the paired-end mode and running 2 × 61 cycles. For quality filtering and removing of remaining adaptor sequences, Trimmomatic-0.39 (62) and a cutoff phred-33 score of 15 were used. The mapping against the reference genomes of *M. maripaludis* JJ (DSM 2067^T^) was performed with Salmon (v 1.5.2) (63). As mapping backbone, a file that contains all annotated transcripts excluding rRNA genes and the whole genome of the reference as decoy was prepared with a k-mer size of 11. Decoy-aware mapping was done in selective-alignment mode with “–mimicBT2,” “–disableChainingHeuristic,” and “–recoverOrphans” flags as well as sequence and position bias correction and 10,000 bootstraps. For –fldMean and –fldSD, values of 325 and 25 were used, respectively. The quant.sf files produced by Salmon were subsequently loaded into R (v 4.0.5) (R Core Team, 2020) using the tximport package (v 1.18.0) (64). DeSeq2 (v 1.30.0) (65) was used for normalization of the reads, and fold change shrinkages were also calculated with DeSeq2 and the apeglm pack-age (v 1.12.0) (66). Genes with a log_2_-fold change of +2/–2 and a *P*-adjust value < 0.05 were considered differentially expressed. Raw reads have been deposited in the Sequence Read Archive (https://www.ncbi.nlm.nih.gov/sra) under accession numbers SRR27061644–SRR27061663 (study number SRP475806).

## SUPPLEMENTAL MATERIAL

Supplemental Material msystems.01338-23-s0001.pdf Tables S1-S10; Figures S1-S8.

ASM does not own the copyrights to Supplemental Material that may be linked to, or accessed through, an article. The authors have granted ASM a non-exclusive, world-wide license to publish the Supplemental Material files. Please contact the corresponding author directly for reuse.
