# Supplementary material for: Proteomic and transcriptomic analysis of selenium utilization in Methanococcus maripaludis
Source: mSystems. 2024 Apr 9;9(5):e01338-23. doi: 10.1128/msystems.01338-23 (PMC11097638; doi:10.1128/msystems.01338-23)
Supplement: Supplemental Material — Tables S1-S10; Figures S1-S8. [file msystems.01338-23-s0001.pdf]

Supplementary Material

Proteomic and transcriptomic analysis of selenium utilization in *Methanococcus maripaludis*

Katrina Funkner, Anja Poehlein, Nico Jehmlich, Richard Egelkamp, Rolf Daniel, Martin von Bergen, and Michael Rother

This PDF file includes:

Supplementary Tables S1 to S10

Supplementary Figures S1 to S8

Supplementary Table S1 – Proteins with higher and lower abundances in selenium free media in comparison to selenium containing media.

LocusTag	Function/Annotation	Fold change	log_2-fold change	Std deviation	p-value
MMJJ_01180	S-layer protein	-4.50	-2.17	0.47	0.01
MMJJ_01210	HemC / Porphobilinogen deaminase	-281.93	-8.14	0.12	9.72×10^{-3}
MMJJ_02140	LolD / ABC-type lipoprotein export system, ATPase component	-46.77	-5.55	N.D.	N.D.
MMJJ_02480	UvrC / Excinuclease UvrABC, nuclease subunit	-3.35	-1.74	0.78	0.02
MMJJ_02830	hypothetical protein	-3.32	-1.73	0.47	2.81×10^{-3}
MMJJ_03300	IlvB / Acetolactate synthase large subunit or other thiamine pyrophosphate-requiring enzyme	-11.00	-3.46	0.79	0.04
MMJJ_03440	hypothetical protein	-3.35	-1.75	0.59	3.63×10^{-4}
MMJJ_05830	Hypothetical protein	-7.82	-2.97	1.2	0.02
MMJJ_05880	S24e / 30S ribosomal protein	-36.53	-5.19	0.75	0.04
MMJJ_06240	Tar / Methyl-accepting chemotaxis protein	-195.75	-7.61	0.86	2.01×10^{-3}
MMJJ_06520	PyrH / Uridylate kinase	-7.40	-2.89	0.73	0.03
MMJJ_07020	Hypothetical protein	-12.30	-3.62	0.4	1.39×10^{-3}
MMJJ_07050	Mtd / F_{420}-dependent methylenetetrahydromethanopterin dehydrogenase	-13.43	-3.75	0.56	7.22×10^{-3}
MMJJ_09170	CcmA / ABC-type multidrucg transport system, ATPase component	-80.62	-6.33	0.088	1.09×10^{-3}
MMJJ_09210	ArsA / Anion-transporting ATPase	-3.46	-1.79	0.56	3.13×10^{-3}
MMJJ_09370	Acetyl-coenzyme A synthetase	-79.77	-6.32	0.51	9.32×10^{-3}
MMJJ_10010	L21e / 50S ribosomal protein	-53.43	-5.74	0.26	1.16×10^{-3}
MMJJ_10210	ArgG / Argininosuccinate synthase	-28.82	-4.85	0.71	0.04
MMJJ_10390	ThiH / 2-iminoacetate synthase	-370.72	-8.53	0.15	2.32×10^{-3}
MMJJ_10890	DNA cytosine methyltransferase	-14.28	-3.84	0.6	6.99×10^{-3}
MMJJ_11320	HdrA2 / Heterodisulfide reductase, subunit A	-134.46	-7.07	0.37	1.60×10^{-3}
MMJJ_11370	VhuB / F_{420}-non-reducing hydrogenase, subunit beta	-3.10	-1.63	0.47	0.05
MMJJ_11380	FwuB / Formyl-methanofuran dehydrogenase	-1695.27	-10.73	0.16	4.25×10^{-4}
MMJJ_12510	Agmatinase	-6.82	-2.77	0.58	2.13×10^{-3}
MMJJ_12780	McrG / Methyl-coenzyme M reductase, subunit gamma	-100.13	-6.65	0.14	8.37×10^{-3}

MMJJ_13340	PorB / Pyruvate synthase subunit Beta	-10.30	-3.36	0.32	2.59x10^{-3}
MMJJ_14230	L32e / 50S ribosomal protein	-59.68	-5.90	0.38	4.03x10^{-3}
MMJJ_14360	L22 / 50S ribosomal protein	-3.77	-1.91	0.75	0.04
MMJJ_14570	FruA / F_{420}-reducing hydrogenase, subunit alpha	-8.34	-3.06	0.27	5.85x10^{-3}
MMJJ_14710	S7 / 30S ribosomal protein	-4.13	-2.04	0.5	7.23x10^{-3}
MMJJ_15150	S9 / 30S ribosomal protein	-33.53	-5.07	0.6	0.05
MMJJ_15440	FdhA1 / F_{420}-dependent formate dehydrogenase, subunit alpha	-4.05	-2.02	N.D.	N.D.
MMJJ_15460	FdhB1 / F_{420}-reducing formate dehydrogenase, subunit beta	-4.61	-2.20	0.47	4.72x10^{-6}
MMJJ_15970	FhcA / Formyltransferase/hydrolase complex	-3.80	-1.93	0.54	1.58x10^{-3}
MMJJ_16020	ThiF / thiamine biosynthesis protein	-4.15	-2.05	0.47	4.51x10^{-3}
MMJJ_16220	Mmp14 / Methanogenesis marker 14 protein	-4.58	-2.19	0.25	2.80x10^{-4}
MMJJ_16640	Iron ABC transporter substrate-binding protein	-5.89	-2.56	0.51	9.28x10^{-4}
MMJJ_17780	RNA-binding domain-containing protein	-37.48	-5.23	0.38	4.05x10^{-3}

MMJJ_00160	ModA / Periplasmic molybdate binding protein	+14.62	+3.87	N.D.	N.D.
MMJJ_01150	POP5 / RNase MRP subunit	+3.13	+1.64	0.97	2.17x10^{-3}
MMJJ_01710	FwdB / formylmethanofuran dehydrogenase subunit B	+5.50	+2.46	0.27	3.43x10^{-4}
MMJJ_01730	VhcB / F_{420}-non-reducing hydrogenase, subunit beta	+520.30	+9.02	N.D.	N.D.
MMJJ_01720	HdrA / Heterodisulfide reductase, subunit A	+310.90	+8.28	0.16	6.92x10^{-3}
MMJJ_01740	VhcA / F_{420}-non-reducing hydrogenase, subunit alpha	+284.01	+8.15	0.16	1.17x10^{-4}
MMJJ_01750	VhcG / F_{420}-non-reducing hydrogenase, subunit gamma	+347.00	+8.44	0.11	4.48x10^{-6}
MMJJ_01760	VhcD / F_{420}-non-reducing hydrogenase, subunit delta	+41.40	+5.37	0.22	3.31x10^{-4}
MMJJ_01770	FrcA / F_{420}-reducing hydrogenase, subunit alpha	+4.00	+2.00	0.51	6.25x10^{-3}
MMJJ_01790	FrcG / F_{420}-reducing hydrogenase, subunit gamma	+13.75	+3.78	N.D.	N.D.
MMJJ_01800	FrcB / F_{420}-reducing hydrogenase, subunit beta	+74.52	+6.22	0.096	1.07x10^{-3}
MMJJ_02740	MnmC / tRNA U34 5-methylaminomethyl-2-thiouridine-forming methyltransferase	+4.97	+2.31	0.76	0.02
MMJJ_02980	Upt / Uracil phosphoribosyltransferase	+2.72	+1.44	0.51	0.01
MMJJ_03000	Predicted transcriptional regulator	+3.47	+1.80	0.92	0.02
MMJJ_03270	T5orf172 domain protein	+3.50	+1.81	0.58	5.38x10^{-3}
MMJJ_03570	CmkB / Cytidylate kinase	+9.86	+3.30	0.6	0.03

MMJJ_03790	Krr1 / rRNA processing protein, contains KH domain	+2.85	+1.51	0.64	0.01
MMJJ_03950	Radical SAM superfamily enzyme with C-terminal helix-hairpin-helix motif	+4.41	+2.14	0.61	$3.45x10^{-4}$
MMJJ_04990	Hypothetical protein	+2.86	+1.52	0.37	$1.54x10^{-3}$
MMJJ_05260	site-specific DNA-methyltransferase (adenine-specific)	+3.26	+1.70	0.99	0.03
MMJJ_05730	SrmB / Superfamily II DNA and RNA helicase	+3.75	+1.91	0.42	$4.54x10^{-4}$
MMJJ_06450	PurD / Phosphoribosylamine-glyvine ligase	+5.49	+2.46	0.17	0.04
MMJJ_06590	PolB / DNA polymerase elongation subunit beta	+2.96	+1.57	0.54	$5.20x10^{-3}$
MMJJ_06980	BsuBI/PstI restriction endonuclease HTH domain	+2.56	+1.36	0.78	0.01
MMJJ_08430	hypothetical protein	+3.40	+1.76	0.52	0.02
MMJJ_09510	ThrC / Threonine synthase	+2.99	+1.58	0.72	0.03
MMJJ_09600	HcgA / Radical S-adenosyl methionine (SAM) enzyme family	+3.10	+1.63	0.69	$9.80x10^{-3}$
MMJJ_09780	AfuA / ABC Fe^{3+} transport system // PgtC / Phosphoglycerate transport regulatory protein PgtC precursor	+19.12	+4.26	0.13	$1.40x10^{-4}$
MMJJ_09980	ARO8 /DNA-binding transcriptional regulator, MocR family, contains an aminotransferase domain	+2.58	+1.37	0.62	0.01
MMJJ_11170	HTH-type transcriptional regulator CysL	+2.76	+1.46	0.79	$6.67x10^{-3}$
MMJJ_11940	SepF / cell division protein	+3.62	+1.86	0.57	0.03
MMJJ_12070	Arginine transport ATP-binding protein ArtM	+2.80	+1.48	0.82	0.04
MMJJ_12610	6-carboxyhexanoate--CoA ligase	+3.14	+1.65	0.72	0.02
MMJJ_13410	HcgB / Guanylyltransferase	+3.91	+1.97	0.55	0.03
MMJJ_13550	ATP-binding cassette domain-containing protein	+2.82	+1.49	0.56	$3.78x10^{-3}$
MMJJ_14030	FtsZ / cell division protein FtsZ	+3.29	+1.72	0.48	0.02
MMJJ_14180	L15P / 50S ribosomal protein	+5.35	+2.42	N.D.	N.D.
MMJJ_14210	L18P / 50S ribosomal protein	+3.57	+1.84	0.77	0.02
MMJJ_14290	L24P / 50S ribosomal protein	+7.35	+2.88	0.34	$3.83x10^{-4}$
MMJJ_14310	S17 / 30S ribosomal protein	+3.45	+1.79	0.78	$1.21x10^{-4}$
MMJJ_14790	DNA-directed RNA polymerase subunit H	+3.22	+1.69	0.77	0.02
MMJJ_15110	hypothetical protein	+3.59	+1.84	0.33	$8.94x10^{-3}$
MMJJ_15350	class I SAM-dependent rRNA methyltransferase	+4.10	+2.04	0.72	$3.03x10^{-3}$
MMJJ_15400	Selenium binding protein	+10.04	+3.33	0.67	$4.86x10^{-3}$
MMJJ_16010	4Fe-4S binding protein	+2.62	+1.39	0.41	$3.05x10^{-3}$

MMJJ_17330	TrmB family transcriptional regulator sugar-binding domain-containing protein	+2.92	+1.54	0.78	0.03
MMJJ_18160	SPFH domain-containing protein	+3.00	+1.58	0.34	3.43×10^{-4}
MMJJ_18360	(R)-citramalate synthase	+3.62	+1.86	0.6	0.04

N.D.: not detected ≥three times in C0 or C8.

Supplementary Table S2 – Raw proteomic data for Sec and Cys-containing isoforms and coexpressed proteins. C0, selenium adequate; C8, selenium depleted; see Materials and Methods.

Protein	C0_1	C0_2	C0_3	C0_4	C0_5	C8_1	C8_2	C8_3	C8_4	C8_5
FwdB	1252601	1860824	3658778	2215745	1755832	9531824	30963569	14904820	8099054	10040169
HdrA	2697391	615013	964415	787959	1422803	209483068	401951789	287985847	170157034	280637809
VhcB	19370	ND	ND	ND	ND	4290979	25026959	8525822	7712479	19804816
vhcA	2024103	529181	628509	461214	724248	179154425	313833402	221171842	105496388	159465476
VhcG	399735	214349	159897	254062	403454	70928186	205588459	92899159	47920552	125889704
VhcD	1069074	895905	1136386	806056	1773900	11196102	57035836	67992742	27935688	45466268
FrcA	709914	323004	363161	194116	503638	1246726	2382571	1736285	1127669	1836174
FrcG	ND	ND	20623	ND	ND	279455	334574	349498	141702	270248
FrcB	ND	15445	ND	23763	33405	1730767	3108813	1799813	1099551	2124708
HdrA2	29333070	18955777	25393786	46603685	23960149	5305930	21988755	6007078	6689628	8770161
VhuD	17144207	24058021	30351202	65549934	69645907	12827080	25238734	11963123	14921526	27332386
VhuG	57588149	22044058	17161329	64287407	33940581	13555851	71936904	14139689	12392522	68892844
VhuA	57826189	43543090	56110514	52666669	63086577	30341013	50356396	49816949	28106073	39108500
VhuB	96653	150171	102518	186938	160263	ND	2621	1196	ND	ND
FwuB	55103932	36508901	55675261	77716131	85573252	18302453	49468679	23396273	21747654	26738176
FruG	732465	1604293	1157624	339626	3919398	6332926	5411880	951728	209594	84939
FruA	71137846	48298381	55980363	50459269	74340983	7150139	11453228	7257213	6734758	7506656
FdhA1	287193	19313	122652	263986	143255	ND	ND	ND	ND	32155
FdhB1	44157499	24551940	35801025	40913960	47070723	13441297	25845991	15590016	15830730	21073562

ND: not detected

Supplementary Table S3 – Genes with higher and lower mRNA abundances in selenium free media in comparison to selenium containing media

LocusTag	Annotation	Fold change	log_2-fold change	Std. deviation	p_{adj}
MMJJ_00160	ModA / Periplasmic molybdate binding protein	55.33	5.79	0.20	$2.42x10^{-191}$
MMJJ_00170	ModB / Membrane protein	14.12	3.82	0.37	$1.67x10^{-24}$
MMJJ_00180	ModC / ATPase	8.51	3.09	0.35	$7.17x10^{-19}$
MMJJ_01600	HgdB / R-phenyllactate dehydratase beta subunit	4.59	2.20	0.13	$4.80x10^{-62}$
MMJJ_01610	SauU/ putative sulfoacetate transporter SauU // UhpC / sugar phosphate permease // MFS / Major Facilitator Superfamily	78.79	6.30	0.13	0
MMJJ_01620	UroD / Uroporphyrinogen decarboxylase	28.64	4.84	0.27	$3.22x10^{-71}$
MMJJ_01630	YejR / GTPase, G3E family	16.56	4.05	0.16	$1.38x10^{-137}$
MMJJ_01640	Uncharacterized 2Fe-2 and 4Fe-4S clusters-containing protein, contains DUF4445 domain	26.35	4.72	0.12	0
MMJJ_01650	SdmC / Methyltransferase	48.17	5.59	0.16	$2.77x10^{-258}$
MMJJ_01660	SdmB / Methyltransferase	54.19	5.76	0.12	0
MMJJ_01670	SdmA / Corrinoid protein	222.86	7.80	0.33	$1.36x10^{-123}$
MMJJ_01710	FwdB / formylmethanofuran dehydrogenase subunit B	608.87	9.25	0.19	0
MMJJ_01720	HdrA / Heterodisulfide reductase, subunit A	1,031.12	10.01	0.20	0
MMJJ_01730	VhcB / F_{420}-non-reducing hydrogenase, subunit beta	1,845.76	10.85	0.22	0
MMJJ_01740	VhcA / F_{420}-non-reducing hydrogenase, subunit alpha	1,937.53	10.92	0.21	0
MMJJ_01750	VhcG / F_{420}-non-reducing hydrogenase, subunit gamma	4,124.49	12.01	0.31	0
MMJJ_01760	VhcD / F_{420}-non-reducing hydrogenase, subunit delta	14,362.31	13.81	0.99	$1.90x10^{-50}$
MMJJ_01770	FrcA / F_{420}-reducing hydrogenase, subunit alpha	803.41	9.65	0.21	0
MMJJ_01780	FrcD / F_{420}-reducing hydrogenase, subunit delta	826.00	9.69	0.35	$6.95x10^{-166}$
MMJJ_01790	FrcG / F_{420}-reducing hydrogenase, subunit gamma	448.82	8.81	0.21	0
MMJJ_01800	FrcB / F_{420}-reducing hydrogenase, subunit beta	315.17	8.30	0.18	0
MMJJ_04340	HisM / ABC-type amino acid transport system, permease component	14.93	3.90	0.39	$7.80x10^{-24}$

MMJJ_04350	HisJ / ABC-type amino acid transport/signal transduction system periplasmic component/ domain // Arginine-binding extracellular protein ArtP precursor	24.42	4.61	0.18	$6.62x10^{-148}$
MMJJ_05450	hypothetical protein	21.86	4.45	2.31	0.00037846
MMJJ_06840	hypothetical protein	8.51	3.09	1.03	0.00066045
MMJJ_07100	HgdB / 2-hydroxyglutaryl-CoA dehydratase subunit	57.28	5.84	0.10	0
MMJJ_07110	PstS / ABD-type phosphate transport system, periplasmic component	6.63	2.73	0.20	$1.58x10^{-40}$
MMJJ_07490	TusD / Sulfurtransferase, subunit delta	24.25	4.60	0.14	$2.19x10^{-242}$
MMJJ_07500	TusA / Sulfurtransferase, subunit alpha	22.47	4.49	0.23	$2.02x10^{-81}$
MMJJ_09590	Hmd / H_2-dependent methylenetetrahydromethanopterin dehydrogenase	37.53	5.23	0.15	$1.62x10^{-253}$
MMJJ_09600	HcgA / Radical *S*-adenosyl methionine (SAM) enzyme family	18.90	4.24	0.10	0
MMJJ_09610	HcgG / FeGP cofactor biosynthesis protein, fibrillarin family	14.42	3.85	0.09	0
MMJJ_09620	Membrane metal-dependend hydrolase	4.69	2.23	0.11	$4.65x10^{-83}$
MMJJ_09720	Xylose isomerase-like TIM barrel	44.32	5.47	0.36	$9.10x10^{-50}$
MMJJ_09730	hypothetical protein	4.32	2.11	0.14	$6.65x10^{-48}$
MMJJ_09740	ApgM / 2,3-bisphosphoglycerate-independent phosphoglycerate mutase	6.77	2.76	0.13	$4.15x10^{-107}$
MMJJ_09770	FbpB / ABC-type Fe^{3+} transport system, permease component	17.51	4.13	0.26	$8.16x10^{-55}$
MMJJ_09780	AfuA / ABC Fe^{3+} transport system // PgtC / Phosphoglycerate transport regulatory protein PgtC precursor	58.49	5.87	0.15	0
MMJJ_09790	EcfT / Energy-coupling factor transporter transmembrane protein	18.13	4.18	0.42	$7.41x10^{-23}$
MMJJ_09800	Ecf / ECF transporter, substrate-specific component	13.09	3.71	0.18	$3.10x10^{-92}$
MMJJ_12990	DUF3343 domain-containing protein	4.86	2.28	0.19	$2.66x10^{-32}$
MMJJ_13000	YedE / YedE family protein	38.85	5.28	0.21	$5.11x10^{-135}$
MMJJ_13010	YedE / YedE family protein	42.22	5.40	0.24	$7.00x10^{-108}$
MMJJ_13020	OB-fold nucleic acid binding domain protein	14.42	3.85	0.25	$6.43x10^{-51}$
MMJJ_13030	TctA / Tripartite tricarboxylate transporter family protein	6.19	2.63	0.55	$1.29x10^{-06}$
MMJJ_14180	50S ribosomal protein L15P	4.17	2.06	0.14	$8.55x10^{-48}$
MMJJ_14190	50S ribosomal protein L30P	4.69	2.23	0.13	$2.17x10^{-63}$
MMJJ_16940	Corrinoid Protein	230.72	7.85	0.42	$1.19x10^{-75}$

MMJJ_16950	Corrinoid Protein	182.28	7.51	0.56	1.09×10^{-38}
MMJJ_16960	Methyltransferase (uroporphyrinogen decarboxylase family protein)	70.52	6.14	0.17	1.19×10^{-300}
MMJJ_18470	TrpC / Indole-3-glycerol phosphate synthase	4.14	2.05	0.17	3.43×10^{-32}

MMJJ_00280	Aquaporin family protein	-13.18	-3.72	0.15	7.67×10^{-128}
MMJJ_00290	DUF2180 family protein	-9.71	-3.28	0.15	1.07×10^{-110}
MMJJ_00300	DUF2193 domain-containing protein	-11.00	-3.46	0.16	7.70×10^{-104}
MMJJ_01140	hypothetical protein	-4.96	-2.31	0.30	4.63×10^{-15}
MMJJ_01380	NifK / Dinitrogenase reductase, component 1, beta chain	-4.56	-2.19	0.21	1.35×10^{-25}
MMJJ_01390	NifD / Dinitrogenase reductase, component 1, alpha chain	-5.54	-2.47	0.19	7.88×10^{-39}
MMJJ_01400	NifI2 / Nitrogen regulatory protein P-II	-11.31	-3.50	0.28	4.56×10^{-36}
MMJJ_01410	NifI1 / Nitrogen regulatory protein P-II	-9.38	-3.23	0.29	2.42×10^{-29}
MMJJ_01420	NifH / Dinitrogenase reductase, component 2	-10.48	-3.39	0.28	7.18×10^{-34}
MMJJ_01490	hypothetical protein	-4.38	-2.13	0.14	4.19×10^{-53}
MMJJ_02240	hypothetical protein	-11.96	-3.58	0.16	7.22×10^{-112}
MMJJ_02940	IbpA / Molecular chaperone, Hsp20 family	-7.01	-2.81	0.21	1.41×10^{-41}
MMJJ_03550	MopI / Molybdopterin-binding protein	-9.99	-3.32	0.22	1.78×10^{-51}
MMJJ_03840	Class III signal peptide	-5.03	-2.33	0.12	2.47×10^{-87}
MMJJ_05410	hypothetical protein	-4.99	-2.32	0.40	4.86×10^{-9}
MMJJ_05800	NifX / Predicted Fe-Mo cluster-binding protein, NifX family	-5.03	-2.33	0.18	6.12×10^{-38}
MMJJ_05810	MinD / MinD superfamily P-loop ATPase, contains ferredoxin domain	-4.41	-2.14	0.18	1.28×10^{-33}
MMJJ_05830	hypothetical protein	-14.83	-3.89	0.28	2.56×10^{-43}
MMJJ_05840	NifX / Predicted Fe-Mo cluster-binding protein, NifX family	-8.34	-3.06	0.19	1.04×10^{-58}
MMJJ_05970	DUF378 containing protein	-5.66	-2.50	0.20	1.44×10^{-36}
MMJJ_05990	Predicted metal dependent hydrolase	-4.23	-2.08	0.12	4.96×10^{-66}
MMJJ_07040	hypothetical protein	-7.01	-2.81	0.16	6.60×10^{-70}
MMJJ_07080	YaaH / YaaH family inner membrane protein	-7.16	-2.84	0.17	2.43×10^{-64}
MMJJ_07520	PorA / Pyruvate:ferredoxin oxidoreductase, subunit alpha	-5.31	-2.41	0.15	1.19×10^{-60}
MMJJ_08070	HesB-like protein	-4.72	-2.24	0.20	3.05×10^{-29}

MMJJ_08460	hypothetical protein	-4.41	-2.14	0.26	$2.84x10^{-16}$
MMJJ_08690	hypothetical protein	-4.96	-2.31	0.88	0.00359068
MMJJ_08710	hypothetical protein	-11.88	-3.57	0.19	$3.77x10^{-82}$
MMJJ_09030	hypothetical protein	-10.56	-3.40	0.55	$7.99x10^{-10}$
MMJJ_09370	Acetyl-coenzyme A synthetase	-6.06	-2.60	0.18	$5.33x10^{-45}$
MMJJ_09710	NfnB / Nitroreductase	-5.13	-2.36	0.14	$1.16x10^{-62}$
MMJJ_10320	GlnB / Nitrogen regulatory protein P-II	-5.94	-2.57	0.24	$3.31x10^{-27}$
MMJJ_10710	tRNA-Ala	-4.79	-2.26	1.31	0.01851843
MMJJ_11320	HdrA2 / Heterodisulfide reductase, subunit A	-19.56	-4.29	0.16	$7.45x10^{-156}$
MMJJ_11330	VhuD / F_{420}-non-reducing hydrogenase, subunit delta	-18.38	-4.20	0.17	$6.74x10^{-128}$
MMJJ_11340	VhuG / F_{420}-non-reducing hydrogenase, subunit gamma	-16.80	-4.07	0.16	$9.02x10^{-134}$
MMJJ_11350	VhuA / F_{420}-non-reducing hydrogenase, subunit alpha	-13.27	-3.73	0.16	$1.29x10^{-114}$
MMJJ_11360	VhuU / F_{420}-non-reducing hydrogenase, subunit U	-10.41	-3.38	0.28	$7.23x10^{-34}$
MMJJ_11370	VhuB / F_{420}-non-reducing hydrogenase, subunit beta	-12.21	-3.61	0.16	$2.97x10^{-112}$
MMJJ_11380	FwuB / Formyl-methanofuran dehydrogenase	-10.41	-3.38	0.16	$4.43x10^{-99}$
MMJJ_11450	Class III signal peptide	-4.11	-2.04	0.14	$9.22x10^{-49}$
MMJJ_12000	DUF169 domain-containing protein	-6.82	-2.77	0.14	$5.43x10^{-82}$
MMJJ_13230	ThsA / Thermosome subunit alpha	-4.38	-2.13	0.16	$1.13x10^{-39}$
MMJJ_13310	PorG / Pyruvate ferredoxin oxidoreductase, subunit gamma	-4.14	-2.05	0.14	$2.95x10^{-47}$
MMJJ_13580	Cobalt/nickel transport system permease protein	-4.96	-2.31	0.14	$9.60x10^{-58}$
MMJJ_13630	hypothetical protein	-4.99	-2.32	0.15	$1.41x10^{-51}$
MMJJ_13700	Monovalent cation/H+ antiporter subunit E	-4.92	-2.30	0.24	$1.54x10^{-21}$
MMJJ_13910	EhaA / Energy-converting NiFe hydrogenase, subunit alpha	-4.17	-2.06	0.22	$3.47x10^{-21}$
MMJJ_13920	Helix-turn-helix domain protein	-6.06	-2.60	0.14	$2.78x10^{-82}$
MMJJ_14540	FruB / F_{420}-reducing hydrogenase, subunit beta	-46.53	-5.54	0.20	$6.73x10^{-165}$
MMJJ_14550	FruG / F_{420}-reducing hydrogenase, subunit gamma	-40.79	-5.35	0.21	$6.31x10^{-146}$
MMJJ_14560	FruD / F_{420}-reducing hydrogenase, subunit delta	-32.00	-5.00	0.21	$5.62x10^{-126}$
MMJJ_14570	FruA / F_{420}-reducing hydrogenase, subunit alpha	-153.28	-7.26	0.28	$1.36x10^{-151}$
MMJJ_14920	Histone family protein	-6.23	-2.64	0.18	$2.81x10^{-47}$
MMJJ_14930	Mmp4 / Methanogenesis marker protein 4	-4.08	-2.03	0.22	$1.35x10^{-19}$
MMJJ_15100	4Fe-4S binding protein	-4.26	-2.09	0.15	$7.94x10^{-44}$

MMJJ_15400	Selenium binding protein	-6.36	-2.67	0.21	$1.81x10^{-36}$
MMJJ_15410	FdhC / F_{420}-reducing formate dehydrogenase, subunit C (Formate Transport)	-5.35	-2.42	0.18	$1.14x10^{-39}$
MMJJ_16710	Peroxiredoxin	-7.21	-2.85	0.13	$9.34x10^{-100}$
MMJJ_16730	DNA protection during starvation protein	-10.63	-3.41	0.20	$2.96x10^{-68}$
MMJJ_16820	CutA / Divalent-cation tolerance protein, subunit alpha	-4.23	-2.08	0.16	$5.13x10^{-38}$
MMJJ_16840	DsrE family protein	-6.77	-2.76	0.16	$2.60x10^{-65}$
MMJJ_16850	hypothetical protein	-6.06	-2.60	0.16	$3.59x10^{-57}$
MMJJ_16860	Putative ferritin	-6.41	-2.68	0.15	$3.50x10^{-68}$
MMJJ_16870	MTH865-like family protein	-5.50	-2.46	0.16	$5.44x10^{-51}$
MMJJ_16880	Class II SORL domain-containing protein	-6.02	-2.59	0.16	$4.42x10^{-59}$
MMJJ_16890	Carboxymuconolactone decarboxylase family protein	-4.69	-2.23	0.21	$3.08x10^{-25}$
MMJJ_16900	HdrB / CoB-CoM heterodisulfide reductase, subunit beta	-5.35	-2.42	0.15	$6.44x10^{-55}$
MMJJ_16910	HdrC / CoB-CoM heterodisulfide reductase, subunit C	-5.58	-2.48	0.15	$2.93x10^{-62}$
MMJJ_17060	Ferredoxin family protein	-4.08	-2.03	0.73	0.00159189
MMJJ_17500	Phosphate ABC transporter substrate-binding protein	-4.86	-2.28	0.22	$3.97x10^{-24}$
MMJJ_17750	DUF4040 domain-containing protein	-5.50	-2.46	0.27	$1.15x10^{-19}$
MMJJ_17760	Monovalent cation/H+ antiporter subunit G	-8.57	-3.10	0.28	$1.08x10^{-27}$
MMJJ_18380	CBS domain-containing protein	-7.21	-2.85	0.17	$7.05x10^{-64}$
MMJJ_18590	hypothetical protein	-6.28	-2.65	0.20	$4.12x10^{-40}$

Supplementary Table S4 – Raw transcriptomic read data for Sec and Cys-containing isoforms and coexpressed genes. C0, selenium adequate; C8, selenium depleted; see Materials and Methods.

Gene	C0_1	C0_2	C0_3	C0_4	C0_5	C8_1	C8_2	C8_3	C8_4	C8_5
fwdB	36.42	57.00	50.60	57.48	51.82	26995.35	22701.32	32860.19	30539.65	40900.53
hdrA	71.87	52.59	52.25	62.04	65.94	54138.09	47562.51	65573.70	62959.31	88395.67
vhcB	20.69	27.81	32.18	26.58	23.19	41860.79	36182.85	47434.90	47781.80	66033.82
vhcA	34.81	22.50	31.77	30.15	18.90	48634.74	41156.30	54735.32	53842.01	72870.75
vhcG	7.50	3.92	6.56	6.04	7.56	21712.26	20886.54	29385.99	26488.52	35508.25
vhcD	0.88	ND	1.24	ND	ND	5953.23	5798.65	8664.59	7436.65	8773.58
frcA	18.85	16.28	6.38	10.71	14.77	9488.37	9479.06	13007.99	11285.48	12062.18
frcD	3.56	2.46	2.52	8.65	2.05	2875.45	2833.44	3940.50	3216.93	3724.19
frcG	14.14	14.45	13.43	20.48	17.32	6693.39	5971.17	8176.97	7401.08	8014.23
frcB	27.87	26.74	25.36	45.33	37.93	8633.04	8499.93	11652.28	10836.68	12500.02
hdrA2	95203.17	88810.51	96727.96	94818.31	90701.57	4190.99	3676.64	5439.99	4644.89	5648.61
vhuD	11424.15	11364.34	11913.90	11368.16	11266.88	532.92	459.57	657.84	668.34	778.34
vhuG	30252.12	27600.30	28279.45	27468.15	26591.99	1384.17	1283.36	1917.58	1638.24	2051.06
vhuA	39380.69	38152.65	38722.12	37451.89	38448.84	2650.00	2314.46	3062.56	2807.11	3519.11
vhuU	157.78	171.51	199.06	165.42	149.87	9.82	16.27	19.17	13.85	21.34
vhuB	32161.85	31034.71	31800.56	29499.40	30612.25	2238.85	1962.54	2827.31	2437.82	3089.67
fwuB	40997.70	40494.51	40185.85	38338.32	40991.95	3608.09	3166.55	3934.95	3891.95	4508.44
fruB	14390.43	12252.13	10964.48	12787.53	13077.57	215.18	244.24	293.55	254.74	341.56
fruG	11292.71	10345.42	9776.87	10907.54	11015.58	252.90	206.96	236.88	254.44	347.33
fruD	6366.13	5580.61	5417.15	6150.20	5859.52	179.79	149.44	162.42	193.63	225.09
fruA	20968.27	17487.14	17018.42	18838.15	19064.22	115.13	111.75	139.43	97.09	140.42
fdhA1	107237.61	96792.67	101256.16	90437.91	99754.26	58924.40	41033.85	84418.98	57737.25	85336.32
fdhB1	168.42	123.08	190.21	164.31	158.68	140.85	126.61	133.18	148.62	143.58

ND: not detected

Supplementary Table S5 - Utilization of various selenium species in *M. maripaludis*. Selenium species were added to 5 nM, 10 nM, 100 nM, 1 µM, readout is Bla activity in JPhydBla2; the data are those used for Fig. 4A

	**5 nM**		**10 nM**		**100 nM**		**1 µM**	
	Bla activity $[Umg^{-1}]$	Standard derivation	Bla activity $[Umg^{-1}]$	Standard derivation	Bla activity $[Umg^{-1}]$	Standard derivation	Bla activity $[Umg^{-1}]$	Standard derivation
SeCN	0.77	0.02	0.11	0.08	0.10	0.00	0.01	0.00
DMSe	n.d.	n.d.	2.21	0.22	1.54	0.21	0.16	0.08
DMDSe	n.d.	n.d.	0.78	0.00	0.02	0.00	0.00	0.00
MSA	n.d.	n.d.	2.30	0.22	1.96	0.22	0.13	0.07
No selenium	n.d.	n.d.	n.d.	n.d.	n.d.	n.d.	2.50	0.30
Selenite	n.d.	n.d.	n.d.	n.d.	n.d.	n.d.	0.02	0.01

n.d.: not determined

Supplementary Table S6 - Utilization of various selenium species in *M. maripaludis*. Selenium species were added to 1 µM, 5 µM, 7.5 µM, 10 µM, 100 µM, readout is Bla activity in JPhydBla2; the data are those used for Fig. 4B

	**1 µM**		**5 µM**		**7.5 µM**		**10 µM**		**100 µM**	
	Bla activity $[Umg^{-1}]$	Standard derivation	Bla activity $[Umg^{-1}]$	Standard derivation	Bla activity $[Umg^{-1}]$	Standard derivation	Bla activity $[Umg^{-1}]$	Standard derivation	Bla activity $[Umg^{-1}]$	Standard derivation
SecSec	2.82	0.52	1.10	0.03	0.65	0.05	0.02	0.00	0.01	0.00
SeMet	2.43	0.13	n.d.	n.d.	n.d.	n.d.	1.73	0.24	0.24	0.05
Selenate	2.79	0.12	n.d.	n.d.	n.d.	n.d.	5.19	0.03	0.22	0.22
MSec	3.39	0.09	n.d.	n.d.	n.d.	n.d.	0.20	0.04	0.02	0.00
DPDS	1.78	0.11	n.d.	n.d.	n.d.	n.d.	2.21	0.26	3.08	0.49
SeUr	2.97	0.15	n.d.	n.d.	n.d.	n.d.	4.25	0.08	3.74	0.47
No selenium	2.50	0.30	n.d.	n.d.	n.d.	n.d.	n.d.	n.d.	n.d.	n.d.
Selenite	0.02	0.01	n.d.	n.d.	n.d.	n.d.	n.d.	n.d.	n.d.	n.d.

n.d.: not determined

Supplementary Table S7 - Utilization of various selenium species by mutant strains lacking putative transporters; the lowest utilized concentration of the respective selenium species was used for these experiments; the data are those used for Fig. 5

	**JPhydbla2**		**Jpst1**		**JpST2**		**JpST3**		**JpST4**		**JpST5**	
	Bla activity [Umg^{-1}]	Standard derivation	Bla activity [Umg^{-1}]	Standard derivation	Bla activity [Umg^{-1}]	Standard derivation	Bla activity [Umg^{-1}]	Standard derivation	Bla activity [Umg^{-1}]	Standard derivation	Bla activity [Umg^{-1}]	Standard derivation
SeCN	0.11	0.09	0.04	0.04	0.07	0.01	0.57	0.08	0.21	0.03	0.24	0.13
DMSe	0.16	0.08	0.00	0.00	0.00	0.00	0.10	0.01	0.01	0.00	0.12	0.07
DMSDe	0.02	0.01	0.01	0.00	0.07	0.02	0.01	0.01	0.01	0.00	0.01	0.00
SecSec	0.02	0.00	0.21	0.11	0.13	0.02	0.18	0.08	0.01	0.00	0.44	0.05
SeMet	0.24	0.05	0.03	0.04	0.07	0.05	0.09	0.04	0.33	0.06	0.22	0.14
Selenate	0.22	0.22	0.12	0.14	0.07	0.03	0.25	0.11	0.25	0.04	0.47	0.20
MSec	0.20	0.04	0.11	0.02	0.06	0.00	0.16	0.04	0.07	0.02	0.15	0.17
MSA	0.13	0.07	0.16	0.04	0.12	0.04	0.14	0.05	0.13	0.06	0.15	0.08
No selenium	2.87	0.12	2.04	0.62	0.79	0.30	1.13	0.15	3.03	0.53	1.59	0.20
Selenite [1µM]	0.02	0.01	0.12	0.14	0.07	0.08	0.02	0.01	0.00	0.00	0.01	0.00

Supplementary Table S8 - Utilization of SeCN at 10 nM by JpST3 and all JpST3 double mutants; the data are those used for Fig. S7

	**JPhydbla2**		**Jpst3**		**JpST1.3**		**JpST2.3**		**JpST4.3**		**JpST5.3**	
	Bla activity [Umg^{-1}]	Standard derivation	Bla activity [Umg^{-1}]	Standard derivation	Bla activity [Umg^{-1}]	Standard derivation	Bla activity [Umg^{-1}]	Standard derivation	Bla activity [Umg^{-1}]	Standard derivation	Bla activity [Umg^{-1}]	Standard derivation
SeCN	0.11	0.09	0.57	0.08	0.14	0.13	0.08	0.06	0.04	0.03	0.32	0.16

Supplementary Table S*9* - Plasmids[a] used in this study.

Name	Description	Reference
pUC19	Amp^R, High copy cloning vector	(1)
pUC57_FrtNpacuptFrt	AmpR, pUC57 BsaI free with Frt-npac-upt-Frt	Biocat.com
pMMD1	Amp^R, pUC19 carrying Frt-*npac*-*upt*-Frt from pUC57_FrtNpacuptFrt, restriction cloned via BamHI	This study
pMMD1_9760up	Amp^R, pMMD1 carrying with the upstream area of *pgtC* (MMJJ_09760). Cloned via SacI and KpnI.	This study
pMMD1_KO9760-90	Amp^R, pMMD1 carrying the up-/downstream area of *pgtC* (MMJJ_09760-90). Downstream cloned via PaeI and SdaI.	This study
pMMD1_7110up	Amp^R, pMMD1 carrying with the upstream area of *psts 1* (MMJJ_07110). Cloned via SacI and KpnI.	This study
pMMD1_KO7110-00	Amp^R, pMMD1 carrying with the up-/downstream area of *psts 1* (MMJJ_07110-00). Downstream cloned via PaeI and SdaI.	This study
pMMD1_1610up	Amp^R, pMMD1 carrying with the upstream area of *sauU* (MMJJ_01610). Cloned via SacI and KpnI.	This study
pMMD1_KO1610	Amp^R, pMMD1 carrying with the up-/downstream area of *sauU* (MMJJ_01610). Downstream cloned via PaeI and SdaI.	This study
pMMD1_dauAup	Amp^R, pMMD1 carrying with the upstream area of *dauA* (MMJJ_04580). Cloned via SacI and KpnI.	This study
pMMD1_KOdauA	Amp^R, pMMD1 carrying with the up-/downstream area of *dauA* (MMJJ_04580). Downstream cloned via PaeI and SdaI.	This study
pMMD1_yedEup	Amp^R, pMMD1 carrying with the upstream area of *yedE* (MMJJ_13000). Cloned via SacI and KpnI.	This study
pMMD1_KOyedE	Amp^R, pMMD1 carrying with the up-/downstream area of *yedE* (MMJJ_13000-10). Downstream cloned via PaeI and SdaI.	This study
pFlpMM	Amp^R, pUC57 bsaI free carrying a *M. maripaludis* codon optimized Flp gene	Biocat.com

a: sequences are available upon request.

1: Yanisch-Perron C, Vieira J, Messing J. 1985. Improved M13 phage cloning vectors and host strains: nucleotide sequences of the M13mp18 and pUC19 vectors. Gene 33:103-119.

Supplementary Table S10 - Oligonucleotides used in this study.

Name	Sequence (5' – 3')[a]	Description
o_MMJJ09760_up_fwd	cgagctcggcgcgccTGGAGAAGACGTTCC	Deletion of *pgtC*
o_MMJJ09760_up_rev	ggggtaccTGGATTATGAATTTTTGGG	Deletion of *pgtC*
o_MMJJ09790_down_fwd	ccgcctgcaggTATGCATTGTTAAGCCC	Deletion of *pgtC*
o_MMJJ09790_down_rev	acatgcatgcggcgcgCCTGTATACCAATTTTGC	Deletion of *pgtC*
o_MMJJ07110_up_fwd	cgagctcggcgcgccTTCACCCCTTGAAATGG	Deletion of *pstS 1*
o_MMJJ07110_up_rev	ggggtaccGTTGGAACAAATGTACAAAATAATCC	Deletion of *pstS 1*
o_MMJJ07100_down_fwd	ccgcctgcaggTGACGAAAATGTTAAAAAATTCATC	Deletion of *pstS 1*
o_MMJJ07100_down_rev	acatgcatgcggcgcgccGTCCGTATTTATTTTCTATTTTAAGG	Deletion of *pstS 1*
o_MMJJ01610_up_fwd	cgagctcggcgcgccTGAAATATTTTTAATGCATCAGTTCC	Deletion of *sauU*
o_MMJJ01610_up_rev	ggggtaccAAACGTTGTCATAAGATCTCC	Deletion of *sauU*
o_MMJJ01610_down_fwd	ccgcctgcaggTTAGGGGGATACACAAGTGC	Deletion of *sauU*
o_MMJJ01610_down_rev	acatgcatgcggcgcgccCTCAACTGCAAGAATTGG	Deletion of *sauU*
o_MMJJ04580_up_fwd	cgagctcggcgcgccTTGCATGGCGTTCTTTTTCG	Deletion of *dauA*
o_MMJJ04580_up_rev	ggggtaccGTGAAACTCCTGAAGCTATTGC	Deletion of *dauA*
o_MMJJ04580_down_fwd	ccgcctgcaggTAAATTTGAAAAATACGGCC	Deletion of *dauA*
o_MMJJ04580_down_rev	acatgcatgcggcgcgccAAAAAACAATTGTAAAAATATTAATGG	Deletion of *dauA*
o_MMJJ13000_up_fwd	cgagctcggcgcgccTTTTTGGTTTTATGTAGTGG	Deletion of *yedE*
o_MMJJ13000_up_rev	ggggtaccAAACCAAACTGCTATGG	Deletion of *yedE*
o_MMJJ13100_down_fwd	ccgcctgcaggACTTACTTTAAAGATAACTTCC	Deletion of *yedE*
o_MMJJ13100_down_rev	acatgcatgcggcgcgccAGAATAAATTATTAATCATTTAATCCC	Deletion of *yedE*

a: restriction sites (and overhangs) for cloning are indicated in lower case.

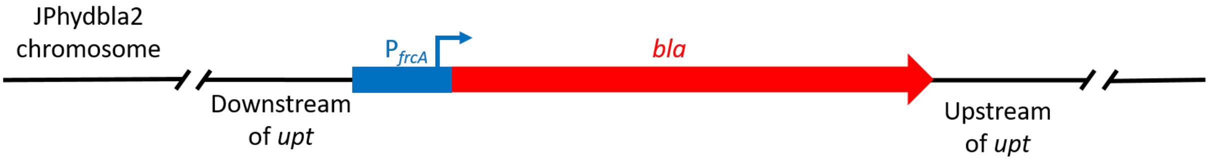

Supplementary Figure S1 – Scheme of the genomic organization of selenium responsive reporter strain JPhydbla2. Blue indicates the *frcA* promoter, red indicates the for *M. maripaludis* codon optimized *bla* gene. Both have been cloned into the *upt* gene of *M. maripaludis*.

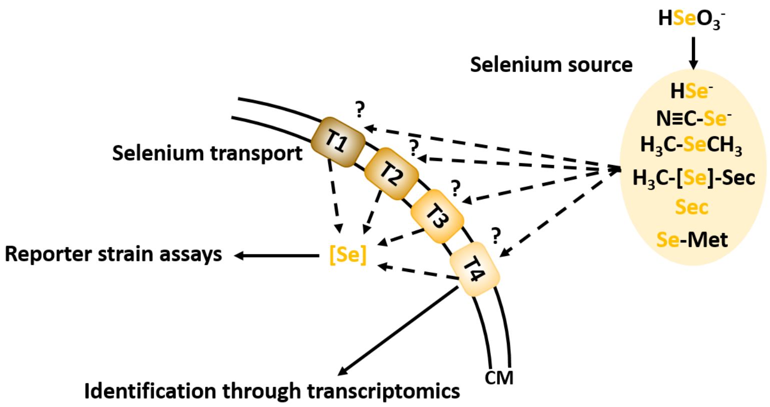

Supplementary Figure S2 - Experimental setup for selenium source and transporter identification

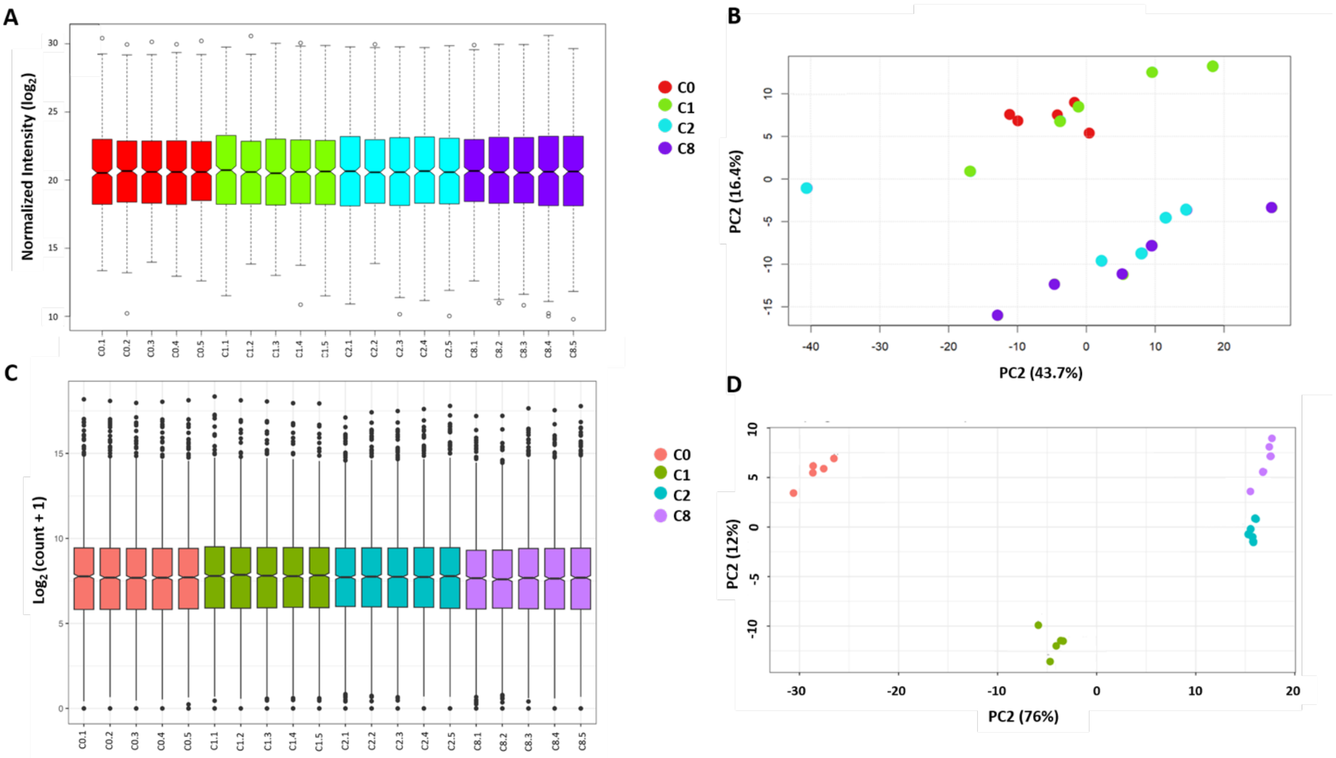

Supplementary Figure S3 – **A** Boxplots of the proteome data for quality control; displayed are the normalized and log_2 transformed intensities measured for each sample. **B** PCA Plot of the proteome data for grouping control; basis for the calculation was the normalized and log_2 transformed intensities measured for each sample. **C** Boxplots of the transcriptome data for quality control; displayed are the normalized and log_2 transformed read counts for each sample. **D** PCA Plot of the transcriptome data for grouping control; basis for the calculation was the normalized and log_2 transformed read counts for each sample.

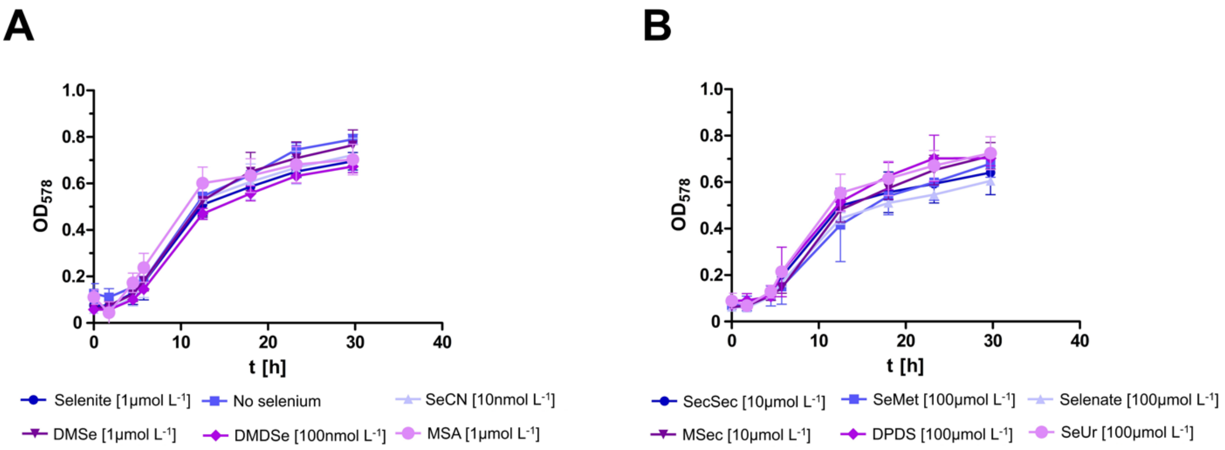

Supplementary Figure S4 - Growth of *M. maripaludis* JPhydbla2 in the presence of various selenium species at their lowest effective concentration (see Fig. 4). **A** Selenium species utilized between 10 nmol l^{-1} – 1 µmol l^{-1}; 1 µmol l^{-1} selenite (blue circle), without selenium (blue square), 10 nmol l^{-1} SeCN (blue triangle), 1 µmol l^{-1} DMSe (pink triangle), 100 nmol l^{-1} DMDSe (pink diamond), 1 µmol l^{-1} MSA (pink circle). B - Selenium species utilized between 10 – 100 µmol l^{-1}; 10 µmol l^{-1} SecSec (blue circle), 100 µmol l^{-1} SeMet (blue square), 100 µmol l^{-1} Selenate (blue triangle), 10 µmol l^{-1} MSec (pink triangle), 100 µmol l^{-1} DPDS (pink diamond), 100 µmol $^{-1}$ SeUr (pink circle). Shown are average values including the standard deviation as error bars from at least three biological replicates.

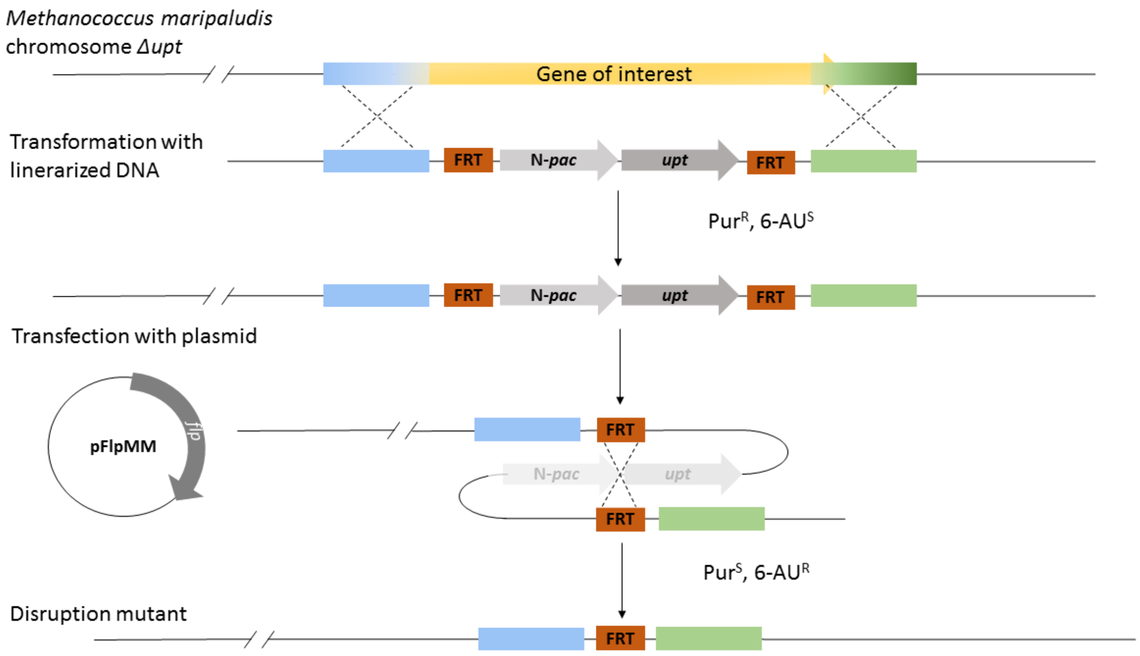

Supplementary Figure S5 - Schematic overview of the markerless disruption method. Linerarized DNA carrying a FRT-N-*pac*-*upt*-FRT cassette (FRT – *flp* recombinase recognition target) as well as the up (blue boxes) and downstream (green boxes) region of the gene of interest (yellow box) is brought into an *M. maripaludis* Δ*upt* strain. Through a double homologous recombination event, the gene of interest is replaced by the FRT-N-*pac*-*upt*-FRT cassette. In a subsequent transfer of a plasmid carrying a codon optimized *flp* recombinase (pFlpMM) for *M. maripaludis* the N-*pac*, *upt* and one FRT side are eliminated by a single homologous recombination event. N-*pac*, gene for puromycin N-acetyl transferase, *upt*, gene for uracil phosphoribosyltransferase, *flp*, gene for *flp* recombinase.

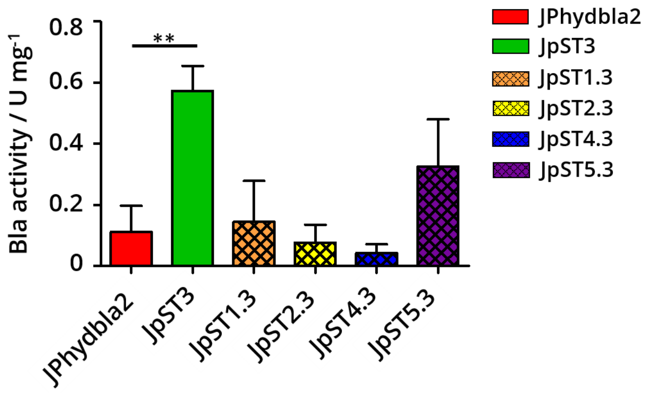

Supplementary Figure S6 – Utilization of SeCN at 10 nmol l^{-1} by JpST3 and all JpST3 double mutants. JPhydbla2 (red bars) was used as the wild type. JpST3 (green bars), JpST1.3 (orange dotted bars), JpST2.3 (yellow dotted bars), JpST4.3 (blue dotted bars) and JpST5.3 (purple dotted bars) have been grown for 16-20 generations on the respective selenium species before testing. An unpaired two-tailed t test was performed comparing the various mutants with JPhydbla2. ** $P < 0.01$, not significant is not shown; shown are average values including the standard deviation as error bars from three biological replicates; the result shown was independently reproduced at least once.

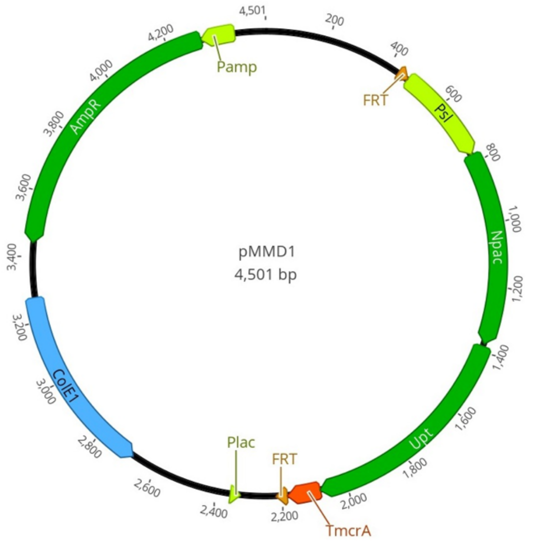

Supplementary Figure S7 - Plasmid map of pMMD1. In light green all three promotors are marked (P*sl*, P*amp*, P*lac*), dark green indicated coding genes (N-*pac, upt, amp*^{R}), red identifies a terminator (TmcrA), orange shows repeat regions (FRT) and blue the ori (ColE1).

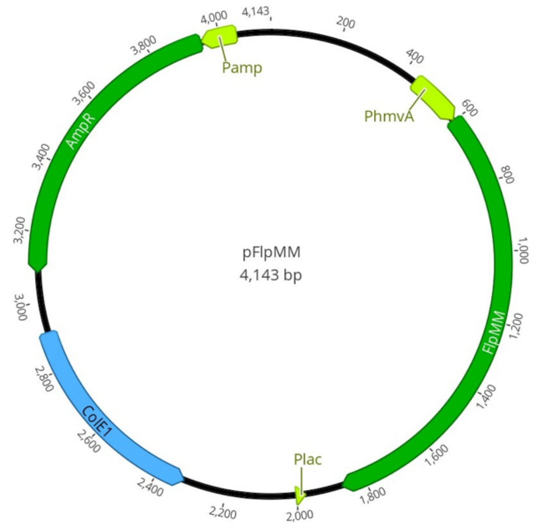

Supplementary Figure S8 - Plasmid map of pFlpMM. In light green, promotors are marked, dark green indicates coding genes (*flpMM*, *amp*^{R}) and blue the ori (ColE1).
